# Respiratory health outcomes in adolescents who vape: a cross-sectional study with biochemically validated vaping status

**DOI:** 10.1136/bmjresp-2026-004463

**Published:** 2026-11-09

**Authors:** Julian Jakob, Yasmin Salem, Vera Van der Velpen, Claudia E Kuehni, Evangelia Liakoni, Maciej L Goniewicz, Rebeca Mozun, Alexander Moeller, Kali Tal, Stéphanie Baggio, Aurélie Berthet, Reto Auer, Philipp Latzin, Sophie Yammine

**Affiliations:** 1 Division of Pediatric Respiratory Medicine and Allergology, Inselspital University Hospital Bern, Bern, Switzerland; 2 Institute of Primary Health Care (BIHAM), University of Bern, Bern, Switzerland; 3 Graduate School for Health Sciences, University of Bern, Bern, Switzerland; 4 Clinical Pharmacology and Toxicology, Department of General Internal Medicine, Inselspital, Bern University Hospital, University of Bern, Switzerland, Inselspital University Hospital Bern, Bern, Switzerland; 5 Institute of Pharmacology, University of Bern, Bern, Switzerland; 6 Institute of Social and Preventive Medicine (ISPM), University of Bern, Bern, Switzerland; 7 Department of Health Behavior, Roswell Park Comprehensive Cancer Center, Buffalo, New York, USA; 8 Department of Intensive Care and Neonatology and Children’s Research Center, University Children’s Hospital Zürich, Zürich, Switzerland; 9 Division of Respiratory Medicine, University Children’s Hospital Zürich, Zurich, Switzerland; 10 Institute of Psychology, University of Lausanne, Lausanne, Switzerland; 11 Centre for Primary Care and Public Health (Unisanté), University of Lausanne, Lausanne, Switzerland

**Keywords:** Tobacco and the lung

## Abstract

**Background:**

The number of vaping adolescents is steadily increasing, but few studies used biochemically validated exposure to assess the effects of vaping on subjective and objective respiratory health outcomes.

**Methods:**

In this cross-sectional analysis (2020–2023) in Switzerland, we included non-vaping adolescents from a birth cohort and vaping adolescents recruited through social media. Exposure categories were self-reported and biochemically validated status of no vaping or smoking in the past year (non-using, reference), vaping or smoking occasionally (occasional use), daily vaping without daily smoking (daily vaping) and daily smoking. Outcomes included respiratory symptoms (longest episode without coughing in the past year), lung function measures (spirometry, Lung Clearance Index), fraction of exhaled nitric oxide and urinary analyses of biomarkers of exposure to respiratory toxicants (polycyclic aromatic hydrocarbons and volatile organic compounds) and biomarkers of oxidative stress. Models were adjusted for sex, age, education level, secondhand smoke exposure, ambient air pollution exposure and atopic disease.

**Results:**

We included 72 participants. Median age was 17 (IQR 16–19) years; 53% (n=38) were women. In multivariable adjusted analyses, daily vaping (n=20) was associated with shorter intervals without coughing (−129 (95% CI −222 to −36) days) and higher exposure to CaHEMA (a metabolite of acrylamide, a respiratory irritant, neurotoxin and probable human carcinogen) compared with non-using adolescents (n=29). We found no difference in lung function outcomes and biomarkers of oxidative stress between all groups.

**Conclusions:**

Although vaping adolescents reported coughing more and had higher acrylamide levels, we detected no difference in lung-function tests. This indicates that respiratory irritation and chemical exposure were present, but functional impairment was not detectable, highlighting the need for longer-term monitoring and more sensitive assessments.

**Trial registration number:**

NCT05105555.

**WHAT IS ALREADY KNOWN ON THIS TOPIC**

- Studies that have shown that adolescents who vape report more respiratory symptoms than their non-vaping peers relied exclusively on self-reported vaping status.

**WHAT THIS STUDY ADDS**

- In this cross-sectional study of 72 participants with biochemically validated exposure, we found that coughing was more often reported by daily vaping adolescents than those who do not vape, but comprehensive lung function outcomes between those two groups did not differ. The study also found that adolescents who vape had elevated exposure to acrylamide (a respiratory irritant, neurotoxin and probable human carcinogen) compared with adolescents who did not vape.

**HOW THIS STUDY MIGHT AFFECT RESEARCH PRACTICE OR POLICY**

- Self-reported respiratory symptoms among adolescents who vape are not reflected in lung function results but are easy to assess. Further objective outcomes and representative longitudinal cohorts starting in childhood are needed to study the long-term health effects of vaping.

## Introduction

 Vaping, also referred to as use of e-cigarettes, is common among adolescents and young adults in industrialised countries. Over 400 000 adolescents report daily vaping in the USA (1.6%) while prevalence is 3.6% in Switzerland.^1 2^ Tobacco is still the most common form of nicotine use in Switzerland (16% daily smokers)^3^ although this proportion has been declining over the years. Some researchers argue that this decline was supported by the introduction of vapes.^4–6^ We are thus observing a shift in nicotine use patterns among adolescents.^7^ Many adolescents who vape report never having smoked tobacco before thus exposing themselves to potential health risks and nicotine addiction.^8^ To estimate the public health effects of vaping we need to better understand the respiratory health outcomes associated with vaping.

Most available data on the somatic health effects of vaping comes from studies in adults. These data differ from data on adolescents and young adults (≤21 years) because adults’ lungs are no longer developing. Those studies also focus on subjective outcomes of health especially in respiratory health such as respiratory symptoms experienced by the user. Most commonly reported respiratory symptoms in adolescents who vape were bronchitis symptoms (cough, phlegm) with an OR between 1.5 and 2.0 for the incidence of these symptoms compared with never vaping adolescents^9–12^ and wheezing with an OR of 1.3–1.4.^13 14^ Very few studies in adolescents reported on the association between vaping and spirometry (traditional lung function testing) and these found no significant difference between those who do and do not vape.^15–17^

Previous studies have four important limitations. First, vaping was defined heterogeneously, with some studies including people who had used any vaping product in the past year, some in the past month and some included daily use. Second, these studies relied on self-reported exposure to vaping and/or smoking but did not validate these reports with markers of tobacco or nicotine exposure (biochemical validation). Third, the presence of smoking-related or vaping-related respiratory symptoms was not complemented by the study of various objective lung function outcomes. Fourth, adolescents who smoke or vape differ vastly from abstaining peers, especially sociodemographically and in exposure to secondhand smoke or other substances, which introduces selection bias unless these characteristics are accounted for.^18^

We thus aimed to study the distribution of subjective (respiratory symptoms) and objective (lung functions and oxidative stress markers) respiratory health outcomes in adolescents and to quantify exposure to inhaled toxicants associated with biochemically validated daily vaping, adjusting for relevant confounders.

## Methods

### Population

We included participants in the Bern-Basel Infant Lung Study (BILD, a Swiss birth cohort study in which few participants reported vaping or smoking)^19^ at their adolescent clinical visit. We separately recruited vaping adolescents through vape shops, schools and social media (primarily Instagram) between 2020 and 2023. Participants were aged 15–21 years and vaped daily, ideally without concurrently smoking tobacco. Because tobacco smoking is highly prevalent in Switzerland (21% of 15–19 year olds currently smoke, 9% smoke daily; 30% of 20–25 year olds smoke, 17% smoke daily),^3^ we expanded our inclusion criteria to ‘possibly no smoking’. We thus included some participants who both smoked and vaped so we could contrast their findings with those of adolescents who vaped exclusively. There were no exclusion criteria.

We obtained written informed consent from each participant. As all participants were 14 years old or older and because the study posed minimal risks for participants, we were not legally required to obtain parental consent according to the Swiss Federal Act on Research involving Human Beings (art. 23). We recorded participants’ age, sex, educational level (secondary or tertiary school education (academic) vs vocational school or labour (labour)) and measured their height, weight, heart rate, systolic, diastolic and mean blood pressure and oxygen saturation at the beginning of each visit. Participants were reimbursed for travel expenses, received snacks and drinks during the 2-hour study visit and were remunerated with a SFr20 CHF voucher for a bookstore or cinema visit.

### Substance use

During the study visit, participants completed a structured online questionnaire on substance use (see the online supplemental appendix for details and the questionnaire).^20 21^ They provided a urinary sample, which we used to assess these biomarkers of exposure: cotinine, a metabolite of nicotine^22^; 4-(methylnitrosamino)-1-(3-pyridyl)-1-butanol (NNAL), a metabolite of tobacco; three metabolites of polycyclic aromatic hydrocarbons (PAH) and 12 metabolites of volatile organic compounds (VOC, see online supplemental appendix for detailed methodology). We used the harmonised acronyms for mercapturic acids.^23^ Cotinine, the main metabolite of nicotine, has a half-life of about 17 hours and is present in people who smoke or vape with nicotine-containing liquids or who have been exposed to these secondhand.^24^ The biomarker NNAL is specific to cigarettes and other tobacco products, has a half-life of several weeks and low clearance and might be present in low amounts even if tobacco exposure ceased months ago.^25^ Liquid chromatography–tandem mass spectrometry (LC-MS/MS) was used to measure cotinine in urine at the clinical pharmacology and toxicology department of General LC-MS lab of the University Hospital Bern (Inselspital), Switzerland.^22^ LC-MS/MS was also used to measure NNAL, metabolites of PAH and VOC in urine at the unit of forensic toxicology and chemistry, University Center of Legal Medicine (Lausanne, Geneva, Switzerland).^26^ We normalised urinary concentrations of cotinine, NNAL, metabolites of PAHs and VOCs to urinary creatinine levels (see online supplemental appendix for detailed methodology).

### Exposure categories

#### Self-reported exposure

First, we built nicotine exposure categories based on self-reports. Participants were classified as non-using (no use of any nicotine product, including tobacco smoking, in the last 12 months), occasional using (vaping or smoking less than daily), daily vaping and daily smoking.

#### Validated exposure

We validated participants’ self-reported exposure with urinary biomarker concentrations of cotinine (nicotine-specific) and NNAL (tobacco-specific).^27^ Based on urinary measures of cotinine, we used the cut-off of 20 ng/mg creatinine to identify active (daily) smoking or vaping adolescents, with reported sensitivity of 97% and specificity of 94%.^28^ Participants who fell below this threshold were considered not to be actively using nicotine; we excluded adolescents from the non-using group if their cotinine levels exceeded the threshold. When the cotinine level of urine exceeded the exposure threshold, we used urinary NNAL to identify active tobacco smoking. We used a cut-off of 10 pg/mg creatinine for NNAL, which identified them as active smokers with a sensitivity and a specificity of 93%.^28 29^ We excluded occasional users if their cotinine or NNAL levels were inconsistent with self-report (eg, they reported no tobacco smoking but their NNAL levels exceeded the threshold). We also excluded adolescents from the daily vaping group if their cotinine levels fell below the threshold or their NNAL levels exceeded the threshold. Finally, we excluded adolescents from the daily smoking group if their NNAL levels fell below the threshold (figure 1).

**Figure 1:**
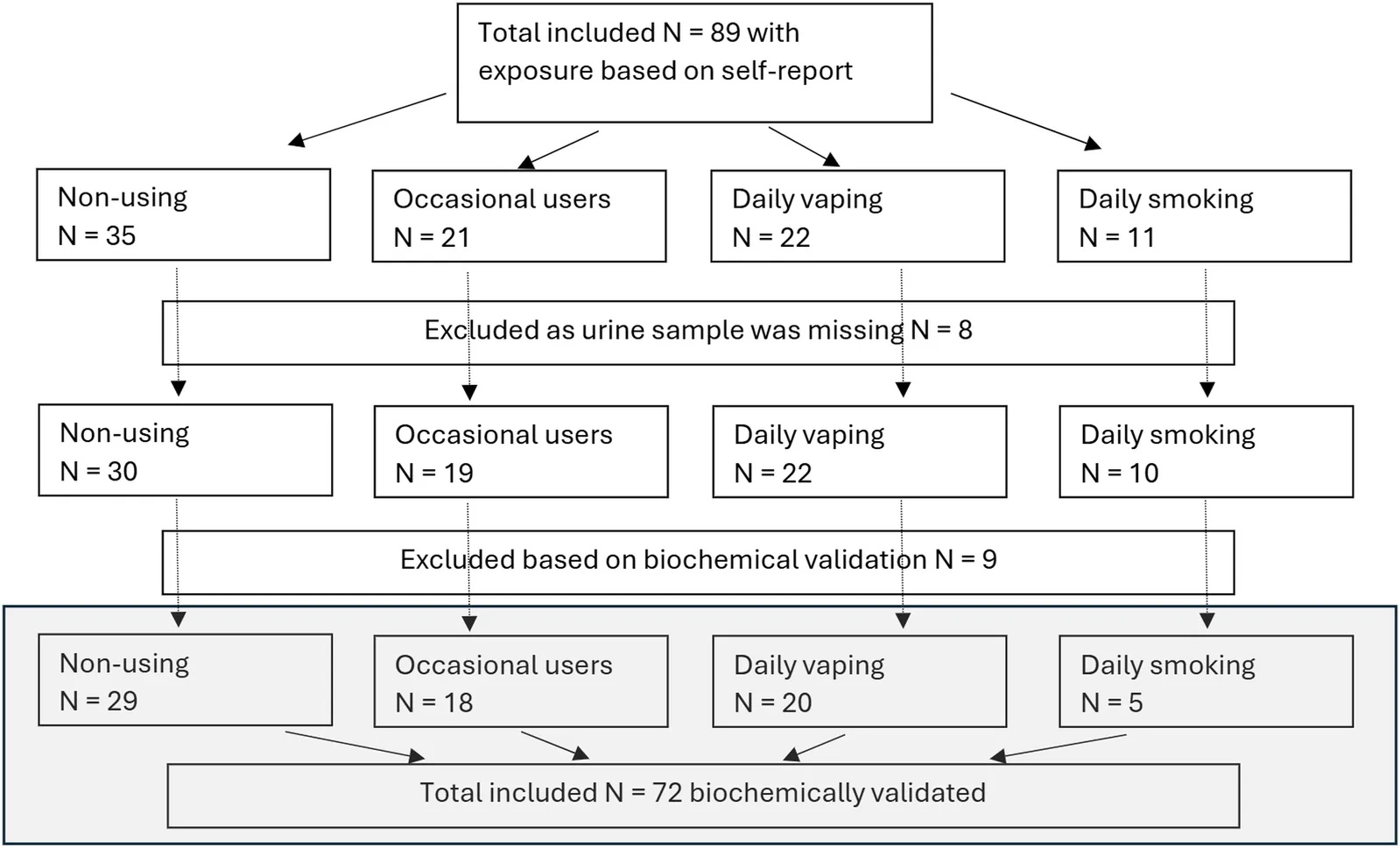
Flow-chart of categorisation of exposure groups. The first row shows the total of adolescents that attended a study visit and their categorisation of exposure based on self-report. Urine samples were missing in eight participants; these participants were excluded. Further, nine participants were excluded because they had discordant exposure categorisation between self-report and biochemical validation.

#### Lab-based exposure

For sensitivity analyses, we added an exposure category based solely on laboratory analyses, without self-report on frequency of exposure. We considered adolescents with cotinine levels below cut-off to be nicotine abstaining, adolescents with cotinine levels above the cut-off and NNAL levels below the cut-off to be active non-tobacco nicotine users and adolescents with NNAL levels above the cut-off to be active tobacco smokers.

#### Biomarkers of exposure to respiratory toxicants

In addition to NNAL and cotinine, which we used to biochemically validate exposures, we assessed biomarkers of exposure to selected respiratory toxicants including three metabolites of PAHs (eg, 1-naphthol, a metabolite of naphthalene) and 12 metabolites of VOCs, including CaHEMA (glycidamide mercapturic acid, a metabolite of acrylamide). For details, see the online supplemental appendix. These markers of exposure to VOC and PAH are elevated in people who smoke cigarettes; prolonged exposure to those VOCs and PAHs is associated with elevated respiratory risk.^30 31^

### Respiratory health outcomes

#### Respiratory symptoms

Participants filled out a digital questionnaire on respiratory symptoms, including the most consecutive days they went without coughing within the past 12 months in days (continuous) and any incidents of wheezing within the past 12 months (binary, both primary respiratory symptom outcomes).^19^

#### Lung function

We assessed lung function with the four following tests: spirometry, body plethysmography, fractional exhaled nitric oxide (FeNO) and multiple-breath washout (MBW). Spirometry provided forced expiratory volume in 1 s (FEV_1_), forced vital capacity (FVC) and the modified Tiffeneau-Pinelli index (FEV_1_/FVC), which we used as measures of overall lung function. We also measured forced expiratory flow between 25% and 75% of FVC (FEF_25–75_) to determine flow in medium to small airways. These measures are routinely used to assess obstructive disorders including asthma.^32^ We performed quality control according to the American Thoracic Society (ATS) and the European Respiratory Society (ERS) criteria and calculated z-scores with Global Lung Initiative (GLI) reference values.^33 34^ Body plethysmography provided measurements of airway resistance, functional residual capacity and total lung capacity. FeNO, a marker of eosinophilic airway inflammation,^35 35^ was measured with a CLD88sp FeNO analyser (ECO MEDICS, Duernten, Switzerland). Following ATS/ERS recommendations, we used the mean of two or three reproducible measurements.^36^ MBW provided the Lung Clearance Index (LCI), which quantifies global ventilation inhomogeneity and serves as a sensitive biomarker of central and peripheral airway function.^37^ Previous studies showed that in people with obstructive lung conditions, LCI is impaired many years before impairment becomes visible in spirometry outcomes. LCI correlates with structural and functional changes observed by using imaging tools.^17^ We defined LCI as the primary lung function outcome and powered the study accordingly (see the Power calculation section below). For LCI by MBW, we used a commercially available, validated setup (Exhalyzer D; Eco Medics AG, Duernten, Switzerland). Data were analysed with Spiroware V.3.3.1 and performed quality control as international guidelines recommended.^38 39^ We used GLI values to calculate z-scores.^40^ LCI values are dimensionless ‘lung turnovers’ and are reported in LCI units.^41^

#### Oxidative stress

We used LC-MS/MS to assess urinary biomarkers of oxidative stress (8-hydroxydeoxyguanosine (8-OHdG) and 8-isoprostane), which, in contrast to the previously mentioned markers of exposure to respiratory toxicants, are markers of effects on the body. Oxidative stress biomarkers have been associated with oxidative DNA damage and cancer.^42 43^

### Power calculation

We aimed to include 21 adolescents who vape daily and 21 who did not vape. Power calculations were based on lung function to contrast daily vaping with non-vaping participants. Since mean LCI in healthy children in previous studies was 6.75±0.57, we determined that including 21 participants in each group would provide 90% power to detect differences in LCI of one population-standard deviation between vaping and non-vaping adolescents, at a two-sided 5% significance level.^44^

### Statistical analyses

All analyses were prespecified: the ClinicalTrials.gov ID is NCT05105555. We used the biochemically validated exposure groups for our main analyses and share results for self-reported and laboratory-based exposure in the online supplemental appendix. We included descriptive results, counts and percentages, mean and SD or median and IQR where appropriate. We described exposure markers and respiratory health measures by exposure category. Then we fitted unadjusted and multivariable-adjusted linear regression models to examine associations between validated exposure categories and biomarkers of exposure to respiratory toxicants and report ß coefficients for these associations. Biomarkers were log-transformed to achieve normality. We analysed respiratory health outcomes similarly, with FeNO log-transformed to achieve normality. In all models, the non-using group was the reference category.^45^

We used logistical regression models for the categorical outcome of wheezing in the past year and present unadjusted and adjusted ORs. We adjusted models for age, sex, educational level, secondhand smoke exposure, self-reported atopic disease and exposure to ambient air pollution (heavy traffic and home oven/chimney use); see online supplemental appendix methods for more information. Whenever adolescents who vaped or smoked daily had higher exposure levels than non-using adolescents or differed from non-users in respiratory health outcomes, we compared daily vaping and daily smoking groups. We report coefficients, Bonferroni corrected 95% CIs and p values from pairwise comparisons of marginal linear predictions.

We used the same multivariable-adjusted models in our sensitivity analyses, which examined the associations between self-reported or laboratory-based exposure categories and the respiratory health outcomes listed above (see online supplemental appendix).

We used Stata MP V.18.0 for all analyses, reported estimates with 95% CIs and used a p value of 0.05 as the level of statistical significance. We adhered to Strengthening the Reporting of Observational Studies in Epidemiology (STROBE) reporting guidelines; see the online supplemental appendix for the detailed checklist.

### Patient and public involvement

Patient and public were not involved in the development of the research questions, outcome measures, study design or study conduct. Results will be shared with study participants, and their perception will be assessed with interviews and/or in focus groups. We will publish a separate report on participants’ perceptions of the results.

## Results

### Population

Our sample totalled 89 participants with self-reported exposures, consecutively recruited from the BILD cohort (non-using adolescents) and separately recruited from the social media campaign. Participants from the campaign were included until we reached 21 vaping adolescents. Although we targeted vaping adolescents, some participants occasionally vaped or smoked or smoked daily. We did not set a target number of people who fell into these latter categories.

Of the 89 enrolled participants, 81 had urinary analyses for exposure markers. After we compared self-declared exposure and biochemical validation, our final sample included 72 participants (table 1): 29 were non-users (all from the BILD cohort, see online supplemental table 1); 18 were occasional users (78% from the BILD cohort); 20 vaped daily (all recruited separately); five smoked daily (60% from the BILD cohort, see figure 1). Median age was 17 years (IQR 16–19), 38 (53%) identified as women, and 39 (54%) reported an academic educational track. Proportions varied widely across nicotine use groups: in the daily vaping group, 75% were women and 45% reported an academic educational track; in the daily smoking group, 60% were women and none reported an academic educational track (see table 1). In the daily vaping group, 80% had ever smoked tobacco; median age at first cigarette was 14 years, median age at first vaping 17 years; 60% reported using disposable vapes; 90% used fruity flavours, and median nicotine concentration was 20 mg/mL (IQR 7–20; for more data, see online supplemental table 1). Agreement between self-reported and biochemically validated exposure was high, Cohen’s kappa=0.75, 80.9% agreement.

**Table 1:** Demographics of the participants using biochemically validated exposure categories

Variable	All participants (N=72)	Non-using* (n=29)	Occasional vaping/smoking (n=18)	Daily vaping (n=20)	Daily smoking (n=5)
Age years, median (Q1; Q3)	17 (16; 19)	16 (16; 17)	17 (16; 17)	19 (18; 20)	17 (17; 20)
Sex, women N (%)	38 (53%)	13 (45%)	7 (39%)	15 (75%)	3 (60%)
Education: secondary or tertiary (academic), N (%)	39 (54%)	23 (79%)	7 (39%)	9 (45%)	0 (0%)
Self-reported exposure to any secondhand smoke, N (%)	26 (36%)	3 (10%)	9 (50%)	11 (55%)	3 (60%)
Oven or fireplace at home, N (%)	29 (40%)	12 (41%)	10 (56%)	5 (25%)	2 (40%)
Trucks driving through street where participant lives, often or very often, N (%)	19 (26%)	8 (28%)	2 (11%)	7 (35%)	2 (40%)
Self-reported atopic disease, N (%)	22 (31%)	10 (34%)	5 (28%)	6 (30%)	1 (20%)
Atopic dermatitis, N (%)	4 (6%)	1 (3%)	2 (11%)	1 (5%)	0 (0%)
Asthma, N (%)	8 (11%)	4 (14%)	3 (17%)	1 (5%)	0 (0%)
Body Mass Index (BMI), kg/m^2^, median (Q1; Q3)	21.5 (19.1; 24.6)	19.8 (18.6; 21.4)	22.3 (20.1; 24.6)	25.3 (21.6; 27.6)	21.5 (20.4; 24.0)
Number of members of household also smoking, median (Q1; Q3)	0 (0; 1)	0 (0; 0)	0 (0; 1)	0 (0; 1)	1 (1; 1)
Quartiles of friends or family members smoking, median (Q1; Q3)†	1 (1; 3)	1 (0; 1)	2 (1; 2)	3 (1; 3)	4 (2; 4)
Quartiles of friends or family members vaping, median (Q1; Q3)†	1 (0; 1)	0 (0; 0)	1 (0; 1)	2 (1; 3)	1 (1; 4)
Self-reported ever vaping, N (%)	42 (58%)	4 (14%)	13 (72%)	20 (100%)	5 (100%)
Years since first vaping in ever vaping, median (Q1; Q3)	1.5 (0.4; 2.6)	0.7 (0.1; 1.7)	1.1 (0.1; 2.6)	2.3 (1.3; 3.6)	1.7 (0.7; 3.5)

* Non-using reported no vaping or smoking in the past 12 months or never vaping/never smoking. Biochemically no current exposure to tobacco and nicotine.

† Quartiles refer to the following proportions: (0) 0%; (1) 1–25%; (2) 26–50%; (3) 51–75%; (4) 76–100%.

Q1, quartile 1; Q3, quartile 3.

### Associations between vaping or smoking and respiratory health symptoms or lung function outcomes

Non-using adolescents reported 180 days without coughing; daily vaping adolescents reported 82 days (table 2). In unadjusted and multivariable adjusted models, daily vaping adolescents reported a shorter episode without coughing in the past 12 months than non-using adolescents did: −129 (95% CI −222 to −36) days (table 3). Wheezing was no more common in daily vaping than in non-using, but it was more common in daily smoking adolescents (online supplemental table 2). FEV_1_ z-score, FVC z-score, FEF_25–75_ z-score, LCI (primary outcome) and log FeNO did not differ between categories. Findings in occasional users resembled those from non-using adolescents.

**Table 2:** Description of respiratory symptoms, lung function measures, oxidative stress metabolites and PAH and VOC metabolites across biochemically validated exposure categories

Variable	All participants (n=72)	Non-using* (n=29)	Occasional vaping/ smoking (n=18)	Daily vaping (n=20)	Daily smoking (n=5)
Respiratory symptoms					
Longest episode without coughing, days, median (IQR)	120 (71 to 240)	180 (98 to 353)	180 (75 to 315)	82 (45 to 101)	120 (14 to 165)
Ever wheezing in the past 12 months, N (%)	10 (14%)	4 (14%)	0 (0%)	2 (10%)	4 (80%)
Lung function, median (IQR)					
FEV1 z-score	−0.5 (−1.0 to 0.5)	−0.4 (−1.0 to 0.4)	−0.5 (−0.9 to 0.7)	−0.7 (−1.2 to 0.1)	−0.3 (−1.8 to 0.8)
FVC z-score	−0.1 (−0.7 to 0.4)	−0.3 (−0.8 to 0.3)	−0.1 (−0.7 to 0.4)	−0.0 (−0.9 to 0.4)	−0.5 (−1.1 to 0.8)
FEV1/FVC %	−0.9 (−1.8 to 2.2)	−0.9 (−1.5 to 2.3)	−0.9 (−1.1 to 2.2)	−0.3 (−2.0 to 2.2)	−1.6 (−1.9 to 2.2)
FEF 25–75 z-score	−0.4 (−1.2 to 0.1)	−0.3 (−1.2 to 0.9)	−0.6 (−1.4 to 0.0)	−0.5 (−1.2 to 0.1)	−0.3 (−1.0 to 0.1)
LCI	6.2 (6.0 to 6.4)	6.4 (5.9 to 6.7)	6.1 (5.9 to 6.4)	6.1 (6.0 to 6.4)	6.2 (6.0 to 6.3)
FeNO, ppb	16.0 (11.8 to 23.2)	14.9 (10.8 to 21.9)	16.4 (13.0 to 23.6)	16.6 (13.0 to 24.5)	16.3 (9.5 to 55.0)
Oxidative stress measures, ng/mg creatinine, median (IQR)†					
OHdG	3.9 (3.0 to 4.6)	3.8 (3.0 to 4.4)	3.9 (2.4 to 5.0)	3.8 (2.4 to 4.9)	4.3 (4.1 to 7.3)
8-Isoprostane	0.2 (0.2 to 0.3)	0.2 (0.2 to 0.3)	0.3 (0.2 to 0.3)	0.2 (0.2 to 0.3)	0.2 (0.2 to 0.4)
Urinary PAH and VOC metabolites, ng/mg creatinine, median (IQR)†					
Polycyclic aromatic hydrocarbons					
1-OHP	0.0 (0.0 to 0.1)	0.0 (0.0 to 0.1)	0.0 (0.0 to 0.1)	0.1 (0.0 to 0.1)	0.1 (0.0 to 0.2)
1-Naphthol	0.3 (0.1 to 0.7)	0.3 (0.1 to 0.3)	0.3 (0.2 to 1.5)	0.2 (0.0 to 0.7)	4.5 (1.1 to 5.3)
2-Naphthol	5.8 (3.4 to 8.1)	6.3 (3.8 to 6.9)	6.2 (3.7 to 7.3)	3.6 (2.2 to 8.6)	10.4 (3.8 to 18.4)
Volatile organic compounds					
CaHEMA	12.1 (7.8 to 18.7)	8.6 (4.2 to 14.7)	9.2 (6.5 to 12.1)	15.8 (12.6 to 27.0)	22.8 (14.0 to 54.8)
2HEMA	1.7 (0.8 to 2.7)	1.9 (1.3 to 3.0)	1.8 (0.7 to 2.5)	1.1 (0.7 to 1.7)	3.0 (2.2 to 5.8)
2CaEMA	44.9 (25.9 to 63.4)	40.6 (24.3 to 58.1)	48.2 (25.9 to 75.6)	33.9 (20.3 to 56.4)	118.1 (83.9 to 171.9)
34HBMA	155.1 (130.0 to 203.9)	174.2 (131.8 to 211.0)	172.5 (140.4 to 200.9)	130.7 (109.9 to 149.1)	277.8 (136.5 to 342.7)
3HPMA	191.2 (110.0 to 381.9)	156.2 (91.0 to 344.9)	222.6 (99.8 to 405.0)	194.2 (149.9 to 281.8)	523.0 (156.4 to 937.0)
2CyEMA	1.3 (0.8 to 4.0)	0.8 (0.4 to 1.2)	1.6 (0.8 to 17.8)	1.7 (1.2 to 3.8)	67.4 (29.4 to 96.0)
2HPMA	21.0 (13.9 to 29.2)	25.4 (16.7 to 29.5)	22.1 (12.7 to 28.7)	15.9 (9.8 to 20.7)	44.8 (20.4 to 48.1)
1HMPeMA	0.0 (0.0 to 0.8)	0.0 (0.0 to 0.0)	0.0 (0.0 to 0.8)	0.7 (0.4 to 0.9)	1.5 (0.0 to 2.2)
2HBeMA	0.0 (0.0 to 0.7)	0.0 (0.0 to 0.0)	0.0 (0.0 to 0.6)	0.6 (0.0 to 0.9)	1.0 (0.5 to 2.0)
4HBeMA	6.3 (3.4 to 8.7)	6.7 (5.5 to 7.5)	7.8 (3.6 to 11.8)	3.2 (1.8 to 6.8)	19.9 (9.0 to 32.6)
3HMPMA	163.4 (129.8 to 215.3)	163.4 (149.4 to 189.1)	225.3 (129.8 to 267.2)	126.9 (103.4 to 165.6)	238.7 (141.5 to 647.2)
PhMA	0.9 (0.6 to 1.5)	1.0 (0.6 to 1.5)	0.7 (0.5 to 1.1)	1.0 (0.6 to 1.6)	1.0 (1.0 to 1.8)

Missings: respiratory symptoms are missing in two participants (one non-using, one occasional using adolescent), spirometry was missing in one participant from the daily vaping group. Urinary markers (oxidative stress, PAH, VOC) are missing in 13 participants (10 non-using, three occasional using adolescents).

* Non-using participants reported no vaping or smoking in the past 12 months or never vaping/never smoking but might be exposed to secondhand smoke.

† Oxidative stress. Policyclic aromatic hydrocarbons (PAH) and volatile organic compounds (VOC, all in ng/mL) in urine normalised to creatinine concentration (mg/mL) in urine (results in ng/mg creatinine).

2CaEMA, 2-carbamoylethylmercapturic acid (a metabolite of acrylamide); CaHEMA, glycidamide mercapturic acid (a metabolite of acrylamide); 2CyEMA, 2-cyanoethylmercapturic acid (a metabolite of acrylonitrile); FEF 25-75, forced expiratory flow between 25% and 75% of forced vital capacity; FeNO, fraction of exhaled nitric oxide; FEV1, forced expired volume in 1 s; FEV1/FVC, modified Tiffeneau-Pinelli index; FVC, forced vital capacity; 34HBMA, dihydroxybutylmercapturic acid (a metabolite of 1,3-butadiene); 2HEMA, 2-hydroxyethylmercapturic acid (a metabolite of ethylene oxide, acrylonitrile or vinyl chloride); 1HMPeMA, 2HBeMA, 4HBeMA, hydroxybutenyl mercapturic acids (metabolites of 1,3-butadiene, 4HBeMA specifically refers to 4-hydroxy-2-buten-1-yl-mercapturic acid); 3HMPMA, 3-hydroxy-1-methyl-propylmercapturic acid (a metabolite of crotonaldehyde); 2HPMA, 2-hydroxypropylmercapturic acid (a metabolite of propylene oxide); 3HPMA, 3-hydroxypropylmercapturic acid (a metabolite of acrolein); LCI, Lung Clerance Index; 1-Naphthol and 2-Naphthol, 1-hydroxynaphthalene and 2-hydroxynaphthalene (metabolites of naphthalene); NNAL, 4-(methylnitrosamino)-1-(3-pyridyl)-1-butanol; 8-OHdG, 8-hydroxydesoxyguanosin; 1-OHP, 1-hydroxypyrene (a metabolite of pyrene); PhMA, phenylmercapturic acid (a metabolite of benzene).

**Table 3:** Unadjusted and adjusted models on the associations between biochemically validated exposure categories and respiratory symptoms, lung function measures, oxidative stress metabolites or PAH and VOC metabolites

Respiratory health measures	Unadjusted, b-coefficient/OR and 95% CI	Fully adjusted, b-coefficient/OR and 95% CI*	Fully adjusted P value†	Comparing daily vaping and smoking (95% CI) and P value‡
Respiratory symptoms				
Longest episode without coughing in days				
Non-using§	Ref	Ref	Ref	
Occasional vaping/smoking	−18 (−83 to 48)	−28 (−98 to 42)	0.43	
Daily vaping	−113 (−176 to −51)	**−129 (−222 to −36**)	**0.008**	Ref
Daily smoking	−104 (−208 to −1)	**−136 (−255 to −18**)	**0.025**	−7 (−160 to 145), p=1
Lung function measures				
FEV1/FVC z-score				
Non-using§	Ref	Ref	Ref	
Occasional vaping/smoking	−0.2 (−1.3 to 1.0)	−0.4 (−1.7 to 0.9)	0.56	
Daily vaping	−0.3 (−1.5 to 0.9)	0.1 (−1.7 to 1.9)	0.94	Ref
Daily smoking	−0.4 (−2.3 to 1.6)	−0.6 (−2.8 to 1.7)	0.63	−0.6 (−3.6 to 2.3), p=1
FEF 25–75 z-score				
Non-using§	Ref	Ref	Ref	
Occasional vaping/smoking	−0.4 (−1.0 to 0.2)	−0.5 (−1.2 to 0.1)	0.12	
Daily vaping	−0.2 (−0.8 to 0.4)	0.0 (-0.9 to 0.9)	0.99	Ref
Daily smoking	−1.0 (−1.1 to 0.8)	−0.2 (−1.4 to 0.9)	0.69	−0.2 (−1.8 to 1.3), p=1
LCI				
Non-using§	Ref	Ref	Ref	
Occasional vaping/smoking	−0.3 (−0.6 to −0.0)	**−0.3 (−0.6 to −0.1**)	**0.014**	
Daily vaping	−0.2 (−0.5 to 0.0)	−0.2 (−0.6 to 0.1)	0.21	Ref
Daily smoking	−0.2 (−0.7 to 0.2)	−0.2 (−0.7 to 0.2)	0.30	−0.0 (−0.6 to 0.6), p=1
Log FeNO¶				
Non-using§	Ref	Ref	Ref	
Occasional vaping/smoking	0.0 (−0.3 to 0.4)	0.0 (−0.4 to 0.4)	0.89	
Daily vaping	0.1 (−0.3 to 0.5)	0.1 (−0.5 to 0.6)	0.82	Ref
Daily smoking	0.3 (−0.4 to 0.9)	0.3 (−0.4 to 0.9)	0.46	0.2 (−0.7 to 1.1), p=1
Oxidative stress metabolites, ng/mg creatinine**	b-coefficient	b-coefficient		
8-OHdG				
Non-using§	Ref	Ref	Ref	
Occasional vaping/smoking	0.3 (−0.7 to 1.3)	0.4 (−0.7 to 1.4)	0.50	
Daily vaping	−0.2 (−1.2 to 0.7)	−0.0 (−1.3 to 1.3)	0.99	Ref
Daily smoking	**1.6 (0.1 to 3.1**)	1.6 (−0.1 to 3.2)	0.066	1.6 (−0.6 to 3.7), p=0.3
PAH/VOC metabolites, ng/mg creatinine**	b-coefficient	b-coefficient		
1-Naphthol				
Non-using§	Ref	Ref	Ref	
Occasional vaping/smoking	0.22 (−0.15 to 0.59)	0.33 (−0.04 to 0.72)	0.078	
Daily vaping	−0.13 (−0.48 to 0.21)	0.27 (−0.20 to 0.74)	0.255	Ref
Daily smoking	**0.92 (0.38 to 1.46**)	**1.34 (0.74 to 1.93**)	**<0.001**	**1.06 (0.30 to 1.83), p=0.002**
CaHEMA				
Non-using§	Ref	Ref	Ref	
Occasional vaping/smoking	0.00 (−0.20 to 0.21)	0.09 (−0.13 to 0.31)	0.42	
Daily vaping	**0.29 (0.09 to 0.48**)	**0.27 (0.00 to 0.54**)	**0.05**	Ref
Daily smoking	**0.48 (0.18 to 0.78**)	**0.55 (0.21 o 0.89**)	**0.002**	0.28 (−0.16 to 0.16), p=0.53
2CyEMA				
Non-using§	Ref	Ref	Ref	
Occasional vaping/smoking	**0.61 (0.27 to 0.94**)	**0.61 (0.26 to 0.97**)	**0.001**	
Daily vaping	**0.44 (0.13 to 0.75**)	0.41 (−0.03 to 0.86)	0.07	Ref
Daily smoking	**1.81 (1.32 to 2.29**)	**1.89 (1.33 to 2.45**)	**<0.001**	**1.48 (0.76 to 2.19), p<0.001**

Bold if p<0.05 for higher exposures.

Missings: respiratory symptoms are missing in two participants (one non-using, one occasional using adolescent), spirometry was missing in one participant from the daily vaping group. Urinary biomarkers (oxidative stress, PAH and VOC metabolites) are missing in 13 participants (10 non-using, three occasional using adolescents). No covariables were missing.

* Linear regression models were adjusted for sex, age, education, exposure to secondhand smoke, oven / chimney at home, heavy traffic near place of living and any atopic disease.

† P values come from a t-test, comparing occasional vaping/smoking, daily vaping or daily smoking to non-using adolescents (reference group).

‡ Contrast estimates, corrected 95% CI and p values come from a pairwise comparisons of marginal linear predictions, applying Bonferroni correction, comparing daily smoking and daily vaping adolescents.

§ Non-using participants reported no vaping or smoking in the past 12 months or never vaping/never smoking but might be exposed to secondhand smoke.

¶ FeNO was log-transformed as its distribution is right skewed.

** Values (ng/mL) in urine normalised to creatinine concentration (mg/mL) in urine (results in ng/mg creatinine). Urinary concentrations of oxidative stress, PAH and VOC metabolites were available for 59 participants (19 (66%) non-using, 15 (83%) occasional users, 20 (100%) daily vaping, five (100%) daily smoking). All other measurements of oxidative stress, PAH and VOC metabolites are presented in online supplemental table 2. All PAH and VOC metabolites were log-transformed to achieve normal distribution.

CaHEMA, glycidamide mercapturic acid (a metabolite of acrylamide); 2CyEMA, 2-cyanoethylmercapturic acid (a metabolite of acrylonitrile); FeNO, fraction of exhaled nitric oxide; FEV1, forced expiratory volume in 1 s; FVC, forced vital capacity; LCI, Lung Clerance Index; OHdG 8, hydroxydesoxyguanosin; PAH, policyclic aromatic hydrocarbons; VOC, volatile organic compound.

### Associations between vaping or smoking and exposure to respiratory toxicants

Urinary analyses of oxidative stress and metabolites of PAH and VOC were available for 59 participants (19 (66%) non-using, 15 (83%) occasional using, 20 (100%) daily vaping and five (100%) daily smoking). Descriptive results of respiratory health outcomes and concentration of exposure biomarkers (metabolites of PAH and VOC) are presented in table 2.

When we compared daily vaping and smoking adolescents with non-using adolescents in multivariable adjusted models, all three of the PAH metabolites we measured were elevated in daily smoking adolescents; none were elevated in daily vaping adolescents. For example, log-transformed 1-Naphthol (PAH metabolite) levels were 1.34 (95% CI 0.74 to 1.93) ng/mg creatinine higher in the daily smoking group than in the non-using group. Levels of log-transformed 1-Naphthol were 1.06 (95% CI 0.3 to 1.83) ng/mg higher in the daily smoking group than in the daily vaping group (table 3). Out of the 12 VOC metabolites we measured, 10 were elevated in the daily smoking group (CaHEMA, 2HEMA, 2CaEMA, 34HBMA, 3–3 HPMA, 2CyEMA, 2HPMA, 1HMPeMA, 3-MHBMA and 3HMPMA) compared with non-using adolescents; five of these were higher in the daily smoking group than in the daily vaping group (2HEMA, 2CaEMA, 2CyEMA, 4HBeMA, 3HMPMA, see online supplemental table 2). One VOC (CaHEMA) was more highly concentrated in the daily vaping group (0.27 (95% CI, 0.00 to 0.54) ng/mg), as was one VOC (2CyEMA) in adolescents who occasionally vaped (table 3, online supplemental table 2, online supplemental figure 1) compared with non-using adolescents.

### Associations between vaping or smoking and exposure to oxidative stress metabolites

Concentrations of urinary oxidative stress biomarkers (8-OHdG and 8-isoprostane) did not differ between categories.

### Sensitivity analyses

When we modelled exposure based only on self-report (n=89; online supplemental table 4 shows demographic data), our results were similar to our analysis of validated exposures for respiratory symptoms and lung function (online supplemental table 4). When we modelled exposure based only on laboratory values (n=81, online supplemental table 5 shows demographic data), our results were similar to the results of our analysis of validated exposures for respiratory symptoms and lung functions; the only difference was that, when we compared both groups to non-using adolescents, 8-OHdG (an oxidative stress biomarker) was elevated in the group of active smokers but not in the group that used nicotine without tobacco (online supplemental table 6).

## Discussion

Adolescents who vaped daily more often reported coughing than non-using adolescents, both in unadjusted models and after multivariable adjustment for sociodemographic variables, exposure to secondhand smoke and ambient air pollution and atopic disease. Spirometry, body plethysmography, LCI, FeNO and urinary oxidative stress markers were similar across groups. Adolescents who vaped had higher urinary concentrations of CaHEMA (a biomarker of exposure to the respiratory irritant, neurotoxin and probable human carcinogen acrylamide^46 47^) than non-using adolescents. Adolescents who smoked or vaped daily reported similar coughing, but their exposure to several PAH and VOC metabolites differed. Our results were consistent whether we categorised exposure groups based on self-report or on laboratory values.

### Respiratory symptoms

Many cross-sectional and longitudinal studies have found that adolescents who vape report respiratory symptoms more often than their non-using peers,^9–11 13 14^ but these studies did not biochemically validate self-reported exposure. We did biochemically validate exposure and found that adolescents who vaped daily reported shorter periods of no cough than non-using adolescents, even after we adjusted for potential confounders.

An earlier study of adults who smoked, but whose respiratory symptoms did not meet the criteria for chronic obstructive pulmonary disease, still reported respiratory symptoms associated with exacerbations that limited their daily activity.^48^ Our findings might thus grow more clinically relevant as the duration of exposure increases. While the respiratory symptoms vaping adolescents reported are likely due to that activity, residual confounding or reporting bias might have influenced our findings.

Non-using participants were drawn from a birth cohort, while participants who vaped were recruited via social media; these groups likely have different sociodemographic characteristics. We thus recommend researchers conduct studies based on representative cohorts that follow children prior to nicotine use, into adolescence and adulthood. Use of such a cohort would allow researchers to track the evolution of these symptoms over time and estimate their potential long-term effects on respiratory health.

### Lung function

In our sample of adolescents, we found no associations between lung function outcomes typically used to assess airway obstruction, for example, FEV_1_ or FEV_1_/FVC z-scores. A study that included 67 vaping adolescents and 1859 controls also found no association between vaping and spirometry measures.^15^ Since a few years of exposure to vaping or tobacco smoking is unlikely to cause larger airway disease by adolescence, we measured the most sensitive marker of ventilation inhomogeneity (LCI assessed by MBW) and found no difference between exposed and unexposed adolescents. A recent Canadian study of otherwise healthy young adults who self-reported their vaping, tobacco smoking, nicotine and cannabis exposure found that LCI was higher among the 41 participants who reported any vaping (mean difference, 0.16 units).^17^ Among vaping participants, 29.3% reported smoking tobacco cigarettes and 51.2% reported concurrent combustible cannabis product use, so findings associated with vaping exposure were confounded. Our data suggest that adolescents who vape or smoke daily, engage in occasional use or are non-users all have similar lung function results, so the value of these markers in estimating the effects of vaping in adolescents with low cumulative exposure to vape or smoke is limited; researchers should conduct studies that follow vaping adolescents over time.

### Oxidative stress

We found no difference in concentrations of urinary oxidative stress biomarkers between our main exposure groups, but when we conducted sensitivity analyses using only laboratory-based exposure, we found higher urinary 8-OHdG (oxidative stress biomarker) concentrations in active smoking adolescents. As a non-specific oxidative stress biomarker, 8-OHdG can be measured after various environmental exposures, including air pollution and tobacco smoke, and may be associated with increased risk of lung and breast cancer.^49^ A study on smoking-naive healthy subjects found that acute vape aerosol inhalation led to a transient increase in oxidative stress and inflammation. Serum markers of oxidative stress and inflammation peaked 1–2 hours post inhalation and returned to baseline after 6 hours.^50^ Two large studies from the USA found no difference in the biomarker concentration of inflammatory or oxidative stress between participants who vaped and non-users but higher levels in tobacco smokers.^51 52^ Our findings are consistent with theirs.

### Limitations

Although we demonstrated that we could recruit vaping adolescents and that collecting a detailed panel of respiratory health outcomes is feasible in this population, we were limited by the fact that participants self-selected their exposure and by their interest level in participating in the study. Control participants from the BILD cohort and vaping or smoking adolescents had a wide range of sociodemographic characteristics, potentially inducing spectrum bias, which we aimed to account for by adjusting for sex, age, education, secondhand smoke exposure and ambient air pollution. Still, the risk of unmeasured confounders remains. Also, participants were asked about respiratory symptoms in the past 12 months, potentially inducing recall or differential reporting bias.

As this study was primarily powered to detect differences in LCI between vaping and non-using participants, our analyses that incorporated other lung function outcomes and that compared daily vaping or smoking adolescents are of limited power. Our findings of no differences between groups should be cautiously interpreted because they may suffer from type II errors. We did not assess heavy metal exposure or exposure to formaldehyde in vaping adolescents, but previous studies did so.^52–54^ Finally, our biochemical validation with cotinine and NNAL did not allow us to differentiate between nicotine vaping and oral nicotine pouch use: we thus relied on self-reports. Nicotine pouch use was minimal: 1/20 (5%) daily vaping adolescents reporting using snus or nicotine pouches at least weekly (online supplemental table 1).

## Conclusions and future implications

Our results varied slightly by exposure category (around 10% exclusion due to conflicting validation compared with self-report). Even though, in our study, self-reported and biochemically validated exposure usually matched, future researchers should consider validating reported exposure. We recommend they conduct prospective studies with long follow-up and comprehensive assessment of exposure to vaping and smoking over time to validate our lung function findings (as ascertained by, eg, FEV_1_, FVC, FEV_1_/FVC, FEF_25–75_, FeNO and LCI). We encourage researchers who want to assess the long-term health effects of vaping to conduct longitudinal studies that start in adolescence and include people with no concurrent tobacco smoking. Our findings lay the groundwork for larger studies with enough statistical power to assess lung function. Researchers could add further biomarkers and objective outcomes to assess the effects of vaping on pulmonary health in this youthful population, for example, MRI, while continuing to collect relevant self-reports on respiratory symptoms.

Adolescents whose self-reported daily vaping was biochemically validated reported more respiratory symptoms than adolescents who did not vape daily, before and after we adjusted for relevant confounders. Vaping adolescents reported coughing more and had higher urinary CaHEMA levels, a biomarker of acrylamide exposure, but we detected no difference in lung-function test results. Longitudinal studies using sensitive measures and repeated assessment of vaping and smoking exposure are needed to determine the long-term respiratory health effects of vaping.

## Supplementary material

10.1136/bmjresp-2026-004463 online supplemental file 1

## Data Availability

Data are available upon reasonable request.
