# Supplementary material for: Respiratory health outcomes in adolescents who vape: a cross-sectional study with biochemically validated vaping status
Source: BMJ Open Respir Res. 2026 Nov 9;13(1):e004463. doi: 10.1136/bmjresp-2026-004463 (PMC13756396; doi:10.1136/bmjresp-2026-004463)
Supplement: Supplementary file 1 [file bmjresp-13-1-s001.docx]

**Biochemically validated vaping and respiratory** **health measures in adolescents. A cross-sectional study.**

Julian Jakob^1,2,3^, Yasmin Salem^1^, Vera van der Velpen^4,5^, Claudia E. Kuehni^6^, Evangelia Liakoni^4^, Maciej Goniewicz^7^, Rebeca Mozun^6,8^, Alexander Moeller^9^, Kali Tal^2^, Stéphanie Baggio^2,10^, Aurélie Berthet^11^, Reto Auer^2,11^, Philipp Latzin^1^, Sophie Yammine^1^

^1^Division of Pediatric Respiratory Medicine and Allergology, Department of Pediatrics, Inselspital, Bern University Hospital, University of Bern, Switzerland

^2^Institute of Primary Health Care (BIHAM), University of Bern, Switzerland

^3^Graduate School for Health Sciences, University of Bern, Switzerland

^4^Clinical Pharmacology and Toxicology, Department of General Internal Medicine, Inselspital, Bern University Hospital, University of Bern, Switzerland

^5^Institute of Pharmacology, University of Bern, Switzerland

^6^Institute of Social and Preventive Medicine (ISPM), University of Bern, Switzerland

^7^Department of Health Behavior, Roswell Park Comprehensive Cancer Center, Elm and Carlton Streets, Buffalo, NY, USA

^8^Department of Intensive Care and Neonatology and Children's Research Center, University Children's Hospital Zurich, University of Zurich, Zurich, Switzerland

^9^Division of Respiratory Medicine, University Children's Hospital Zurich, Zurich, Switzerland

^10^ Institute of Psychology, University of Lausanne, Lausanne, Switzerland

^11^Centre for Primary Care and Public Health (Unisanté), University of Lausanne, Switzerland

Contact Address:

Julian Jakob, MD PhD

Department of Pediatrics, Division of Pediatric Respiratory Medicine and Allergology

Inselspital, Bern University Hospital

Freiburgstrasse 15

3010 Bern

Switzerland

julian.jakob@unibe.ch

ONLINE APPENDIX

Contents

[Appendix Methods	3](#_Toc241167313)

[1.	DAG of the covariables included (SHS = Second-Hand Smoke)	3](#_Toc241167314)

[2.	Content of questionnaire	3](#_Toc241167315)

[3.	Original substance use questionnaire	4](#_Toc241167316)

[4.	Respiratory symptoms questions and confounders	6](#_Toc241167317)

[5.	Detailed method of urinary analyses	6](#_Toc241167318)

[Sensitivity analyses	8](#_Toc241167319)

[Appendix Table 1: Detailed characteristics of participants	9](#_Toc241167320)

[Appendix Table 2: Unadjusted and adjusted models on self-reported and biochemically validated exposure categories and respiratory health measures, and exposure biomarker concentrations, all measures. Linear regression models for continuous outcomes (providing b-estimates), logistic regression models for binary outcomes (providing Odds Ratio OR).	12](#_Toc241167321)

[Appendix Table 3: Demographics of the participants with **self-reported** exposure categories.	18](#_Toc241167322)

[Appendix Table 4: Unadjusted and adjusted models on **self-reported** exposure categories. Linear regression models for continuous outcomes (providing b-estimates), logistic regression models for binary outcomes (providing Odds Ratio OR).	19](#_Toc241167323)

[Appendix Table 5: Demographics of the participants using **lab-based** exposure categories	25](#_Toc241167324)

[Appendix Table 6: Unadjusted and adjusted models with **lab-based** exposure categories. Linear regression models for continuous outcomes (providing b-estimates), logistic regression models for binary outcomes (providing Odds Ratio OR).	26](#_Toc241167325)

[Appendix Figure 1: Multivariable adjusted models on self-reported and biochemically validated exposure categories and respiratory symptoms, lung function markers (FEV1/FVC, LCI), 8-Hydroxydesoxyguanosin (8-OHdG, oxidative stress marker), 1-Naphthol (PAH metabolite, log10 transformed), and three VOC metabolites (CaHEMA, 2CaEMA, 2CyEMA, all log10 transformed)	31](#_Toc241167326)

[STROBE Statement – Filled Checklist	33](#_Toc241167327)

[Appendix Reference	35](#_Toc241167328)

# Appendix Methods

## 1. DAG of the covariables included (SHS = Second-Hand Smoke)

Sex^1^, age, education^4^, SHS exposure^5^, atopic disease^6^

Respiratory health measure

Vaping / smoking

Ambient air pollution (oven, fireplace, heavy traffic)^3^

Socioeconomical status^2^

## 2. Content of questionnaire

We queried information included history and current intensity of vaping or smoking, device used, nicotine type (freebase versus salts), e-liquid nicotine concentration and flavoring, reason for vaping, nicotine dependency measures as queried by the time to first nicotine use in the morning, questions on use of other tobacco and nicotine products, alcohol and drug use, exposure to second-hand smoking, and proportion of friends or family members smoking or vaping.^7,8^

## 3. Original substance use questionnaire

## 4. Respiratory symptoms questions and confounders

Participants filled a digital questionnaire on respiratory symptoms including the longest episode without coughing in the past 12 months in days (continuous), and ever wheezing in the past 12 months (binary, both primary respiratory symptom measures).^9^ Number of episodes of colds, coughing at night or during sports, and mucus expectorations without having a cold in the past 12 months.

Confounders: We assed exposure to ambient air pollution based on participants’ living situation (using an open fireplace or chimney to heat at home, frequency of heavy traffic / trucks driving next to their place of living). We asked participants about the number of hours per week they were exposed to second-hand smoke at home, at work or in their leisure time, and dichotomized this variable into no reported exposure versus any second-hand smoke exposure. Participants reported self-declared respiratory and atopic disease (asthma, atopic dermatitis, allergic rhino-conjunctivitis).

## 5. Detailed method of urinary analyses

Participants provided a urinary sample to study the following markers of exposure: cotinine (a metabolite of nicotine),^10^ NNAL (4-(methylnitrosamino)-1-(3-pyridyl)-1-butanol, a metabolite of tobacco-specific nitrosamines (TSNAs)), three metabolites of polycyclic aromatic hydrocarbons (PAHs): 1-hydroxypyrene (1-OHP; a metabolite of pyrene), 1- and 2-naphthol (two metabolites of naphthalene), and 12 metabolites of volatile organic compounds (VOCs): Glycidamide mercapturic acid (CaHEMA; a metabolite of acrylamide), -carbamoylethylmercapturic acid (2CaEMA; a metabolite of acrylamide), 2-Hydroxyethylmercapturic acid (2HEMA; a metabolite of ethylene oxide, acrylonitrile, or vinyl chloride), 2 dihydroxybutylmercapturic acid (34HBMA; a metabolite of 1,3-butadiene), monohydroxybutenyl-mercapturic acids (1HMPeMA, 2HBeMA, and 4HBeMA; metabolites of 1,3-butadiene, MHBMA-3 specifically refers to 4-hydroxy-2-buten-1-yl-mercapturic acid), 3-hydroxypropylmercapturic acid (3HPMA; a metabolite of acrolein), 2-cyanoethylmercapturic acid (2CyEMA; a metabolite of acrylonitrile), -2 2-hydroxypropylmercapturic acid (2HPMA; a metabolite of propylene oxide), 3-hydroxy-1-methyl-propylmercapturic acid (3HMPMA; a metabolite of crotonaldehyde), and phenylmercapturic acid (PhMA; a metabolite of benzene).

Cotinine is a metabolite of nicotine, with a half-life of 17 hours. It is found in people smoking or vaping with nicotine-containing liquids, or in people with second-hand exposure to these.^11^ TSNAs are specific to tobacco products, including cigarettes, they are thus excellent biomarkers of tobacco exposure, including second-hand smoke. They have a half-live of several weeks and their clearance are low, therefore TSNAs might be found in low amounts even If tobacco exposure has ceased months ago.^12^ Urinary analyses of cotinine were performed using LC-MS/MS at the Clinical Pharmacology and Toxicology LC-MS lab, University Hospital Bern (Inselspital), Switzerland.^10^ Urinary analyses of NNAL and metabolites of PAHs and VOCs, as well as the oxidative stress biomarkers were performed using LC-MS/MS at the Unit of Forensic Toxicology and Chemistry, University Center of Legal Medicine (Lausanne – Geneva, Switzerland).^13^ We normalized urinary concentrations of cotinine, NNAL, the metabolites of oxidative stress, PAHs and VOCs to urinary creatinine levels, quantified with a routine clinical method based on Jaffe on a chemical analyzer (AU480 Chemistry Analyzer, Beckman Coulter, Nyon, Switzerland).

LOQ were 0.5ng/ml for 8-OHdG, 0.1ng/ml for 8-Isoprostane, 0.2ng/ml for PAH metabolites and 2.0ng/ml for VOC metabolites.

LOD were 0.2ng/ml for 8-OHdG, 0.05ng/ml for 8-Isoprostane, 0.1ng/ml for PAH metabolites and 1.0ng/ml for VOC metabolites. Participants’ values <LOQ were imputed as LOQ/2. Participants’ values <LOD were imputed as LOD/2.^14^

**Cotinine**

Urine samples were mixed, centrifuged and subsequently pre-diluted 1:50 in water. For enzymatic hydrolysis, 15 µL of pre-diluted urine was mixed with 15 µL water and 30 µL of a solution containing β-glucuronidase (BGTurbo, Kura Biotech, Atlanta, GA, USA) and deuterated IS. This mixture was tapped to mix, spun down and subsequently diluted with 0.01% NH₄OH in water to a final volume of 210 µL. Fifty microliters of the sample was loaded onto an Oasis HLB solid-phase extraction (SPE) column (15 µm, 2.1 × 20 mm; Waters) at 0% mobile phase B for further online sample clean-up. The concentration of B was increased to 15% within 1 minute. At 2 minutes, analytes were backflushed onto the analytical column with 20% B. Following a 0.2-minute equilibration, mobile phase B increased linearly to 35% at 3 minutes, then held for 0.5 minutes. It was subsequently ramped to 50% at 4.5 minutes. Both columns were washed at 100% B and re-equilibrated, yielding a total run time of 9 minutes. Calibration standards ranged from 1 to 250 ng/mL and curves corresponding to the relevant concentrations in the samples were constructed using at least 6 calibrator points. The lower limits of quantification (LLOQs) for cotinine, was 1 ng/mL in pre-diluted urine, corresponding to 50 ng/mL in undiluted urine due to the initial 1:50 dilution. If analyte concentrations in the 1:50 diluted samples exceeded the upper limit of the calibration range, the samples were re-analyzed using a 1:200 dilution. Conversely, if detectable peaks were observed below the LLOQ, a 1:10 dilution was used to increase analyte concentrations in the injected sample, effectively lowering the LLOQ to 10 ng/mL in the original undiluted urine.

**PAH metabolites (from^13^)**

Three PAH metabolites: 1- and 2-naphthol and 1-OHP, were analyzed with a routine laboratory method. Briefly, urine (3 mL) was mixed with a solution (3 mL) containing internal standards (1-OHP-d9, 1-naphtol-d7, and 2-naphtol-d7), enzyme (β-glucoronidase from [*Helix pomatia*](https://www.sciencedirect.com/topics/pharmacology-toxicology-and-pharmaceutical-science/helix-pomatia) type HP-2 ≥ 100,000 U/mL, Merck KGaA, Buchs, St. Gallen, Switzerland), and [hydrochloric acid](https://www.sciencedirect.com/topics/pharmacology-toxicology-and-pharmaceutical-science/hydrochloric-acid) (4 N) in sodium acetate buffer (32.8 g/L). The mixed solution was incubated at 37 °C overnight. The target analytes were extracted with SPE (ABN 60 mg/3 mL, Biotage, Uppsala, Sweden). The extract was dried and reconstituted in water/acetonitrile (500 μL, 80:20, *v*/v) for LC-MS/MS analysis. Limits of detection (LODs) were 0.1 ng/mL and LOQs were 0.2 ng/mL for each metabolite. A quality control (QC) was injected every 15 samples and the sample series were included if these were within 15 % of the given value. Performance of the method was assessed by inter-laboratory tests (G-EQUAS n°66–67).

**VOC metabolites (from^13^)**

VOC metabolites were quantified with a LC-MS/MS according to the method of [Alwis et al. (2012)](https://www.sciencedirect.com/science/article/pii/S0048969722054602?via%3Dihub#bb0015). Briefly, filtered urine samples were diluted 1:10 with buffer (100 μL urine +50 μL internal standards +850 μL 15 mM [ammonium acetate](https://www.sciencedirect.com/topics/pharmacology-toxicology-and-pharmaceutical-science/ammonium-acetate) pH 6.8). The diluted solution was directly injected in the LC-MS/MS. LOQs were 1.5 ng/mL for 3HPMA, 2HPMA and 3HMPMA, 2 ng/mL for 2CaEMA, 34HBMA, 2CyEMA, 1HMPeMA, 2HBeMA, 4HBeMA and PhMA, and 3 ng/mL for CaHEMA and 2HEMA. A QC sample was injected every 10 samples and the sample series were included if these were within 10 % of the given value (except for MHBMA: 15 %). Performance of the method was assessed by inter-laboratory tests (G-EQUAS n°67–68) for all analytes, except 3HMPMA.

**NNAL (from^13^)**

NNAL was quantified by LC-MS/MS (Acquity UPLC system + Xevo TQ MS, Waters, Baden-Dättwil, Switzerland). Samples were prepared at the department of occupational and environmental health (DSTE) of the Center for Primary Care and Public Health (Unisanté, Lausanne). The method was based on Biotage application note (n°AN884, 2017 ([Biotage, n.d.](https://www.sciencedirect.com/science/article/pii/S0048969722054602?via%3Dihub#bb0050))) and the publications of [Byrd and Ogden (2003)](https://www.sciencedirect.com/science/article/pii/S0048969722054602?via%3Dihub#bb0065), [Kavvadias et al. (2009)](https://www.sciencedirect.com/science/article/pii/S0048969722054602?via%3Dihub#bb0210), and [Hu et al. (2014)](https://www.sciencedirect.com/science/article/pii/S0048969722054602?via%3Dihub#bb0175). Briefly, urine samples (1.3 mL) were centrifuged (4000 rpm, 5 min) and mixed with internal standards (100 μL), buffer (200 μL, phosphate buffer 1 M) and enzyme (400 μL of β-glucoronidase ~5 mg/mL or ~ 5200 U/mL; β-glucoronidase from *Escherichia coli* type IX-A 125KU, Merck KGaA, Buchs, St. Gallen, Switzerland). After one night in the dark at 37 °C, the mixture was extracted with a solid-supported liquid/liquid extraction (SLE cartridge; ISOLUTE® SLE+ 2 mL Sample, Biotage, Uppsala, Sweden) and the samples were analyzed by LC-MS/MS at EPFL. LOQ was 0.2 ng/mL. A QC sample was injected every 15 samples and the sample series were included if these were within 10 % of the given value.

**8-oxodG and 8-isoprostane (from^13^)**

8-oxodG and 8-isoprostane were analyzed with a previously described LC-MS/MS (Dionex Ultimate 3000 system + TSQ Quantiva, Thermo Scientific, Reinach, Switzerland) method ([Sambiagio et al., 2020](https://www.sciencedirect.com/science/article/pii/S0048969722054602?via%3Dihub#bb0315)). Sample preparation included a SPE (Chromabond C18ec SPE 500 mg 3 mL, Macherey-Nagel, Oensingen, Switzerland). LOQs were 0.5 ng/mL for 8-oxodG and 0.1 ng/mL for 8-isoprostane. Two QCs were injected every 15 samples and the sample series were included if these were within 10 % of the given value.

## Sensitivity analyses

When modelling exposure based on self-report only (N=89, Appendix Table 3 shows demographic data), results were similar to results from validated exposures for respiratory symptoms and lung functions. We found 1/3 PAH metabolites were elevated in daily smoking, none in daily vaping adolescents (Figure 2). We found 5/12 VOCs elevated in daily smoking, none in daily vaping or occasionally using adolescents, Appendix Table 4). When modelling exposure based on laboratory values only (N=81, Appendix Table 5 shows demographic data), results were similar to results from validated exposures for respiratory symptoms and lung functions, but 8-OHdG (oxidative stress biomarker) was elevated in active smoking but not in non-tobacco nicotine using compared to nicotine abstaining adolescents (Appendix Table 6). All three metabolites of PAH showed high concentrations in active smoking, but not in non-tobacco nicotine using adolescents compared to nicotine abstaining adolescents. Six out of twelve metabolites of VOC showed high concentrations in active smoking, one in non-tobacco nicotine using compared to nicotine abstaining adolescents (Appendix Table 6).

# Appendix Table 1: Detailed characteristics of participants

| Variable | All participants  (N=72) | Non-using^a^  (n=29) | Occasional vaping / smoking  (n=18) | Daily vaping  (n=20) | Daily smoking (n=5) |
| --- | --- | --- | --- | --- | --- |
| Provenance, N (%):  - From BILD cohort  - Recruited separately | 46 (64%)  26 (36%) | 29 (100%)  0 (0%) | 14 (78%)  4 (22%) | 0 (0%)  20 (20%) | 3 (60%)  2 (40%) |
| Nicotine depend. Score^b^, median (Q1; Q3) | 1 (0; 2) | 0.0 (0; 0) | 0.(0; 0) | 2 (1; 3) | 2 (2; 3) |
| SUBSTANCE USE |  |  |  |  |  |
| Self-reported ever vaping. N (%) | 42 (58%) | 4 (14%) | 13 (72%) | 20 (100%) | 5 (100%) |
| Self-reported current vaping, N (%) |  |  |  |  |  |
| - not in past 12mths | 42 (58%) | 29 (100%) | 10 (56%) | 0 (0%) | 3 (60%) |
| - < monthly | 4 (6%) | 0 (0%) | 4 (22%) | 0 (0%) | 0 (0%) |
| - monthly | 1 (1%) | 0 (0%) | 1 (6%) | 0 (0%) | 0 (0%) |
| - weekly | 4 (6%) | 0 (0%) | 3 (17%) | 0 (0%) | 1 (20%) |
| - daily | 21 (29%) | 0 (0%) | 0 (0%) | 20 (100%) | 1 (20%) |
| Puffs/day in daily vapers, median (Q1; Q3) | 225 (100; 600) | n/a | n/a | 200 (100; 600) | 800 (800; 800) |
| Device type, N (%) |  |  |  |  |  |
| - tanks | 4 (6%) | 0 (0%) | 0 (0%) | 4 (20%) | 0 (0%) |
| - pods | 6 (8%) | 0 (0%) | 2 (11%) | 4 (20%) | 0 (0%) |
| - disposable | 16 (22%) | 0 (0%) | 2 (11%) | 12 (60%) | 2 (40%) |
| Fruity flavoring, N (%) | 27 (38%) | 0 (0%) | 7 (39%) | 18 (90%) | 2 (40%) |
| Tobacco flavoring, N (%) | 1 (1%) | 0 (0%) | 0 (0%) | 1 (5%) | 0 (0%) |
| Vanilla flavoring, N (%) | 2 (3%) | 0 (0%) | 1 (6%) | 1 (5%) | 0 (0%) |
| Nicotine type, N (%) |  |  |  |  |  |
| - no nicotine | 5 (7%) | 0 (0%) | 5 (28%) | 0 (0%) | 0 (0%) |
| - free-base nicotine | 5 (7%) | 0 (0%) | 0 (0%) | 5 (25%) | 0 (0%) |
| - nicotine salts | 20 (28%) | 0 (0%) | 3 (17%) | 15 (75%) | 2 (40%) |
| Nicotine concentration, mg/ml, median (Q1 ; Q3) | 20 (3; 20) | n/a | 0 (0; 20) | 20 (7; 20) | 20 (20; 20) |
| Age at first vaping, median (Q1; Q3) | 16 (15; 18) | 145 (15; 16) | 15 (14; 16) | 17 (15; 19) | 15 (14; 17) |
| Years vaping, median (Q1; Q3) | 1.5 (0.4; 2.6) | 0.7 (0.1; 1.7) | 1.1 (0.1; 2.6) | 2.3 (1.3; 3.6) | 1.7 (0.7; 3.5) |
| Reason for vaping: Trying something new, N (%) | 16 (22%) | 2 (7%) | 8 (44%) | 4 (20%) | 2 (40%) |
| Smoking cessation, N (%) | 8 (11%) | 0 (0%) | 0 (0%) | 6 (30%) | 2 (40%) |
| For fun, N (%) | 21 (29%) | 0 (0%) | 7 (39%) | 12 (60%) | 2 (40%) |
| Because a friend vapes, N (%) | 10 (14%) | 0 (0%) | 5 (28%) | 4 (20%) | 1 (20%) |
| Self-reported ever tobacco smoking | 35 (49%) | 2 (7%) | 12 (67%) | 16 (80%) | 5 (100%) |
| Self-reported current smoking |  |  |  |  |  |
| - not in past 12mths | 46 (64%) | 29 (100%) | 7 (39%) | 10 (50%) | 0 (0%) |
| - < monthly | 11 (15%) | 0 (0%) | 4 (22%) | 7 (35%) | 0 (0%) |
| - monthly | 5 (7%) | 0 (0%) | 4 (22%) | 1 (5%) | 0 (0%) |
| - weekly | 5 (7%) | 0 (0%) | 3 (17%) | 2 (10%) | 0 (0%) |
| - daily | 5 (7%) | 0 (0%) | 0 (0%) | 0 (0%) | 5 (100%) |
| Cigarettes/day in daily smokers | 9 (4; 10) | n/a | n/a | n/a | 9 (4; 10) |
| Age at first smoking, years median (Q1; Q3)  Range | 15 (14; 16)  12-19 | 16 (16; 16)  16-16 | 15 (15; 16)  15-19 | 14 (13; 16)  12-19 | 15 (14; 15)  13-15 |
| Years smoking, median (Q1; Q3) | 2.6 (1.3; 4.4) | 0.8 (0.1; 1.4) | 1.4 (0.5; 2.1) | 4.2 (3.0; 5.6) | 3.9 (2.6; 5.3) |
| Other tobacco / nicotine forms (weekly-daily) |  |  |  |  |  |
| Hooka, N (%) | 4 (6%) | 0 (0%) | 0 (0%) | 3 (15%) | 1 (20%) |
| Cigars, N (%) | 1 (1%) | 0 (0%) | 1 (6%) | 0 (0%) | 0 (0%) |
| Pipe, N (%) | 0 (0%) | 0 (0%) | 0 (0%) | 0 (0%) | 0 (0%) |
| Snus / nicotine pouches, N (%) | 4 (6%) | 0 (0%) | 2 (11%) | 1 (5%) | 1 (20%) |
| Cannabis smoking weekly or more, N (%) | 8 (11%) | 0 (0%) | 2 (11%) | 2 (10%) | 4 (80%) |
| Further illicit drugs weekly or more^c^, N (%) | 3 (4%) | 0 (0%) | 0 (0%) | 2 (10%) | 1 (20%) |
| Alcohol drinking weekly or more, N (%) | 20 (28%) | 2 (7%) | 6 (33%) | 7 (35%) | 5 (100%) |
| Binge drinking weekly or more, N (%)^d^ | 2 (3%) | 0 (0%) | 0 (0%) | 1 (5%) | 1 (20%) |
| Anthropometrics |  |  |  |  |  |
| Height, cm, median (Q1; Q3) | 171.7 (165.0; 178.4) | 174.0 (165.4; 179.4) | 177.4 (169.2; 180.6) | 170.2 (161.6; 177.8) | 165.4 (161.9; 170.1) |
| Weight, kg, median (Q1; Q3) | 63.4 (55.0; 73.2) | 59.0 (51.5; 63.7) | 70.5 (59.8; 74.4) | 70.2 (56.6; 85.0) | 59.0 (54.7; 67.8) |
| Heart rate, median (Q1; Q3) | 78 (69; 85) | 77 (67; 85) | 78 (68; 86) | 81 (75; 85) | 75 (64; 88) |
| Systolic blood pressure, mmHg, median (Q1; Q3) | 119 (112;127) | 121 (113;128) | 118 (108; 134) | 120 (115; 124) | 112 (110; 120) |
| Diastolic blood pressure, mmHg, median (Q1; Q3) | 73 (66; 79) | 72 (66; 82) | 71 (65; 81) | 76 (69; 80) | 62 (58; 77) |
| Oxygen saturation, %, median (Q1; Q3) | 98 (98; 99) | 98 (98; 99) | 98 (97; 100) | 98 (97; 99) | 98 (98; 99) |
| Respiratory symptoms (continued) |  |  |  |  |  |
| Number of episodes of colds, median (IQR) | 2 (2; 5) | 2 (2; 2) | 2 (2; 2) | 2 (2; 5) | 5 (2; 7) |
| Dry coughing at night in the past 12 months, N (%) | 18 (25%) | 2 (7%) | 4 (22%) | 9 (45%) | 3 (60%) |
| Coughing during sports in the past 12 months, N (%) | 26 (36%) | 5 (17%) | 7 (39%) | 9 (45%) | 5 (100%) |
| Mucus expectoration without a cold in the past 12 months, N (%) | 5 (7%) | 1 (3%) | 1 (6%) | 2 (10%) | 1 (20%) |

a Abstainers reported no vaping or smoking in the past 12 months or never vaping / never smoking

b Queried by the question “How fast do you vape / smoke in the morning?" Answers were <5 minutes (4 points), 6 to 30 minutes (3 points), 31-60 minutes (2 points), >60 minutes (1 point), and no daily use (0 points).

c Cocaine, 3,4-Methylenedioxymethamphetamine (MDMA), Ketamine, lysergic acid diethylamide (LSD), magic mushrooms. Nobody reported opioids use

d Defined as having more than 4 (men) or 3 (women) standard drinks on a drinking episode

# Appendix Table 2: Unadjusted and adjusted models on self-reported and biochemically validated exposure categories and respiratory health measures, and exposure biomarker concentrations, all measures. Linear regression models for continuous outcomes (providing b-estimates), logistic regression models for binary outcomes (providing Odds Ratio OR).

| **Respiratory health measures** | **Unadjusted, b-coefficient / OR and 95 % CI** | **Fully adjusted, b-coefficient / OR and 95 % CI^b^** | **Fully adjusted p-value^c^** | **Comparing daily vaping and smoking (95% CI) and p-value^d^** |
| --- | --- | --- | --- | --- |
| **RESPIRATORY SYMPTOMS** |  |  |  |  |
| **Longest episode without coughing in days** | **b-coefficient** | **b-coefficient** |  |  |
| Non-using^a^ | Ref | Ref | Ref |  |
| Occasional vaping / smoking | -18 (-83 to 48) | -28 (-98 to 42) | 0.43 |  |
| Daily vaping | -113 (-176 to -51) | **-129 (-222 to -36)** | **0.008** | Ref |
| Daily smoking | -104 (-208 to -1) | **-136 (-255 to -18)** | **0.025** | -7 (-160 to 145), p=1 |
| **Ever wheezing in the past 12 months (yes/no)** | **Odds Ratio** | **Odds Ratio** |  |  |
| Non-using^a^ | Ref | Ref | Ref |  |
| Occasional vaping / smoking | n/a | n/a | n/a |  |
| Daily vaping | 0.7 (0.1 to 4.0) | 1.1 (0.0 to 45.4) | 0.94 | Ref |
| Daily smoking | 24.0 (2.1 to 274) | **111 (1.2 to >1000)** | **0.043** | 4.5 (-0.8 to 10.0), p=0.16 |
| **Number of episodes of cold in the past 12 months** | **b-coefficient** | **b-coefficient** |  |  |
| Non-using^a^ | Ref | Ref | Ref |  |
| Occasional vaping / smoking | 0 (-1 to 2) | 0 (-1 to 2) | 0.87 |  |
| Daily vaping | 2 (1 to 4) | **2 (0 to 5)** | **0.023** | Ref |
| Daily smoking | 2 (0 to 5) | 2 (-1 to 5) | 0.11 | -0 (-4 to 3), p=1 |
| **Dry coughing at night in the past 12 months (yes/no)** | **Odds Ratio** | **Odds Ratio** |  |  |
| Non-using^a^ | Ref | Ref | Ref |  |
| Occasional vaping / smoking | 4.0 (0.6 to 24.8) | 5.4 (0.6 to 47.8) | 0.13 |  |
| Daily vaping | 10.6 (2.0 to 57.4) | 12.9 (1.0 to 173.6) | 0.054 | Ref |
| Daily smoking | 19.5 (2.0 to 193.6) | **20.3 (1.1 to 383.6)** | **0.044** | 0.5 (-3.1 to 4.0), p=1 |
| **Coughing during sports in the past 12 months (yes/no)** | **Odds Ratio** | **Odds Ratio** |  |  |
| Non-using^a^ | Ref | Ref | Ref |  |
| Occasional vaping / smoking | 3.3 (0.8 to 13.1) | 3.1 (0.7 to 14.5) | 0.16 |  |
| Daily vaping | 3.4 (0.9 to 12.8) | 5.1 (0.6 to 45.3) | 0.15 | Ref |
| Daily smoking | n/a | n/a | n/a | n/a |
| **Mucus expectoration without a cold in the past 12 months (yes/no)** | **Odds Ratio** | **Odds Ratio** |  |  |
| Non-using^a^ | Ref | Ref | Ref |  |
| Occasional vaping / smoking | 2.7 (0.7 to 9.6) | 2.3 (0.5 to 10.3) | 0.26 |  |
| Daily vaping | 7.0 (1.9 to 25.3) | 2.6 (0.3 to 20.5) | 0.35 | Ref |
| Daily smoking | n/a | n/a | n/a | n/a |
| **LUNG FUNCTION MEASURES** | **b-coefficient** | **b-coefficient** |  |  |
| **FEV1 z-score** |  |  |  |  |
| Non-using^a^ | Ref | Ref | Ref |  |
| Occasional vaping / smoking | 0.2 (-0.4 to 0.8) | 0.2 (-0.4 to 0.9) | 0.46 |  |
| Daily vaping | -0.1 (-0.7 to 0.5) | 0.3 (-0.6 to 1.3) | 0.47 | Ref |
| Daily smoking | -0.1 (-1.1 to 0.9) | 0.0 (-1.1 to 1.2) | 0.95 | -0.3 (-1.8 to 1.2), p=1 |
| **FVC z-score** |  |  |  |  |
| Non-using^a^ | Ref | Ref | Ref |  |
| Occasional vaping / smoking | 0.2 (-0.4 to 0.8) | 0.4 (-0.2 to 1.1) | 0.20 |  |
| Daily vaping | 0.1 (-0.5 to 0.6) | 0.3 (-0.6 to 1.2) | 0.56 | Ref |
| Daily smoking | 0.0 (-0.9 to 1.0) | 0.3 (-0.8 to 1.4) | 0.58 | 0.0 (-1.4 to 1.5), p=1 |
| **FEV1/FVC z-score** |  |  |  |  |
| Non-using^a^ | Ref | Ref | Ref |  |
| Occasional vaping / smoking | -0.2 (-1.3 to 1.0) | -0.4 (-1.7 to 0.9) | 0.56 |  |
| Daily vaping | -0.3 (-1.5 to 0.9) | 0.1 (-1.7 to 1.9) | 0.94 | Ref |
| Daily smoking | -0.4 (-2.3 to 1.6) | -0.6 (-2.8 to 1.7) | 0.63 | -0.6 (-3.6 to 2.3), p=1 |
| **FEF 25-75 z-score** |  |  |  |  |
| Non-using^a^ | Ref | Ref | Ref |  |
| Occasional vaping / smoking | -0.4 (-1.0 to 0.2) | -0.5 (-1.2 to 0.1) | 0.12 |  |
| Daily vaping | -0.2 (-0.8 to 0.4) | 0.0 (-0.9 to 0.9) | 0.99 | Ref |
| Daily smoking | -1.0 (-1.1 to 0.8) | -0.2 (-1.4 to 0.9) | 0.69 | -0.2 (-1.8 to 1.3), p=1 |
| **FRC z-score** |  |  |  |  |
| Non-using^a^ | Ref | Ref | Ref |  |
| Occasional vaping / smoking | 0.3 (-0.3 to 0.8) | 0.3 (-0.3 to 0.9) | 0.30 |  |
| Daily vaping | -0.3 (-0.9 to 0.2) | 0.1 (-0.7 to 1.0) | 0.73 | Ref |
| Daily smoking | -0.1 (-1.1 to 0.8) | 0.1 (-0.9 to 1.1) | 0.81 | -0.0 (-1.3 to 1.3), p=1 |
| **TLC z-score** |  |  |  |  |
| Non-using^a^ | Ref | Ref | Ref |  |
| Occasional vaping / smoking | 0.4 (-0.1 to 0.9) | 0.4 (-0.2 to 0.9) | 0.17 |  |
| Daily vaping | 0.0 (-0.5 to 0.5) | 0.0 (-0.7 to 0.8) | 0.95 | Ref |
| Daily smoking | -0.2 (-0.9 to 0.6) | -0.1 (-1.0 to 0.8) | 0.76 | - 0.2 (-1.4 to 1.1), p=1 |
| **Raw** |  |  |  |  |
| Non-using^a^ | Ref | Ref | Ref |  |
| Occasional vaping / smoking | -0.03 (-0.07 to 0.01) | -0.01 (-0.05 to 0.03) | 0.57 |  |
| Daily vaping | -0.02 (-0.06 to 0.02) | -0.00 (-0.06 to 0.06) | 0.97 | Ref |
| Daily smoking | -0.03 (-0.10 to 0.03) | -0.02 (-0.09 to 0.05) | 0.58 | - 0.02 (-0.11 to 0.07), p=1 |
| **LCI** |  |  |  |  |
| Non-using^a^ | Ref | Ref | Ref |  |
| Occasional vaping / smoking | -0.3 (-0.6 to -0.0) | **-0.3 (-0.6 to -0.1)** | **0.014** |  |
| Daily vaping | -0.2 (-0.5 to 0.0) | -0.2 (-0.6 to 0.1) | 0.21 | Ref |
| Daily smoking | -0.2 (-0.7 to 0.2) | -0.2 (-0.7 to 0.2) | 0.30 | -0.0 (-0.6 to 0.6), p=1 |
| **Log FeNO^e^** |  |  |  |  |
| Non-using^a^ | Ref | Ref | Ref |  |
| Occasional vaping / smoking | 0.0 (-0.3 to 0.4) | 0.0 (-0.4 to 0.4) | 0.89 |  |
| Daily vaping | 0.1 (-0.3 to 0.5) | 0.1 (-0.5 to 0.6) | 0.82 | Ref |
| Daily smoking | 0.3 (-0.4 to 0.9) | 0.3 (-0.4 to 0.9) | 0.46 | 0.2 (-0.7 to 1.1), p=1 |
| **OXIDATIVE STRESS METABOLITES, ng/mg creatinine^f^** | **b-coefficient** | **b-coefficient** |  |  |
| **8-OHdG** |  |  |  |  |
| Non-using^a^ | Ref | Ref | Ref |  |
| Occasional vaping / smoking | 0.3 (-0.7 to 1.3) | 0.4 (-0.7 to 1.4) | 0.50 |  |
| Daily vaping | -0.2 (-1.2 to 0.7) | -0.0 (-1.3 to 1.3) | 0.99 | Ref |
| Daily smoking | **1.6 (0.1 to 3.1)** | 1.6 (-0.1 to 3.2) | 0.066 | 1.6 (-0.6 to 3.7), p=0.3 |
| **8- Isoprostane** |  |  |  |  |
| Non-using^a^ | Ref | Ref | Ref |  |
| Occasional vaping / smoking | 0.00 (-0.06 to 0.06) | 0.01 (-0.06 to 0.07) | 0.86 |  |
| Daily vaping | -0.01 (-0.07 to 0.04) | 0.02 (-0.06 to 0.11) | 0.57 | Ref |
| Daily smoking | 0.05 (-0.04 to 0.14) | 0.07 (-0.03 to 0.18) | 0.16 | 0.05 (-0.08 to 0.18), p=1 |
| **PAH / VOC METABOLITES, ng/mg creatinine^f^** | **b-coefficient** | **b-coefficient** |  |  |
| **1-OHP** |  |  |  |  |
| Non-using^a^ | Ref | Ref | Ref |  |
| Occasional vaping / smoking | -0.05 (-0.28 to 0.19) | -0.02 (-0.27 to 0.024) | 0.90 |  |
| Daily vaping | 0.16 (-0.06 to 0.38) | 0.10 (-0.21 to 0.42) | 0.52 | Ref |
| Daily smoking | **0.51 (0.17 to 0.86)** | **0.54 (0.14 to 0.94)** | **0.01** | 0.44 (-0.07 to 0.99), p=0.13 |
| **1-Naphthol** |  |  |  |  |
| Non-using^a^ | Ref | Ref | Ref |  |
| Occasional vaping / smoking | 0.22 (-0.15 to 0.59) | 0.33 (-0.04 to 0.72) | 0.078 |  |
| Daily vaping | -0.13 (-0.48 to 0.21) | 0.27 (-0.20 to 0.74) | 0.255 | Ref |
| Daily smoking | **0.92 (0.38 to 1.46)** | **1.34 (0.74 to 1.93)** | **<0.001** | **1.06 (0.30 to 1.83), p=0.002** |
| **2-Naphthol** |  |  |  |  |
| Non-using^a^ | Ref | Ref | Ref |  |
| Occasional vaping / smoking | 0.06 (-0.18 to 0.30) | 0.13 (-0.12 to 0.37) | 0.30 |  |
| Daily vaping | -0.01 (-0.23 to 0.21) | 0.03 (-0.27 to 0.33) | 0.83 | Ref |
| Daily smoking | 0.26 (-0.08 to 0.61) | **0.41 (0.03 to 0.79)** | **0.033** | 0.38 (-0.11 to 0.87), p=0.22 |
| **CaHEMA** |  |  |  |  |
| Non-using^a^ | Ref | Ref | Ref |  |
| Occasional vaping / smoking | 0.00 (-0.20 to 0.21) | 0.09 (-0.13 to 0.31) | 0.42 |  |
| Daily vaping | **0.29 (0.09 to 0.48)** | **0.27 (0.00 to 0.54)** | **0.05** | Ref |
| Daily smoking | **0.48 (0.18 to 0.78)** | **0.55 (0.21 o 0.89)** | **0.002** | 0.28 (-0.16 to 0.16), p=0.53 |
| **2HEMA** |  |  |  |  |
| Non-using^a^ | Ref | Ref | Ref |  |
| Occasional vaping / smoking | -0.11 (-0.30 to 0.71) | -0.07 (-0.26 to 0.13) | 0.49 |  |
| Daily vaping | **-0.22 (-0.39 to -0.05)** | -0.11 (-0.36 to 0.13) | 0.34 | Ref |
| Daily smoking | 0.22 (-0.04 to 0.49) | **0.38 (0.08 to 0.68)** | **0.02** | **0.49 (0.10 to 0.88), p=0.006** |
| **2CaEMA** |  |  |  |  |
| Non-using^a^ | Ref | Ref | Ref |  |
| Occasional vaping / smoking | 0.01 (-0.20 to 0.23) | 0.05 (-0.16 to 0.27) | 0.62 |  |
| Daily vaping | -0.08 (-0.28 to 0.12) | 0.07 (-0.20 to 0.34) | 0.58 | Ref |
| Daily smoking | **0.45 (0.14 to 0.77)** | **0.64 (0.30 to 0.98)** | **<0.001** | **0.57 (0.13 to 1.00), p=0.005** |
| **34HBMA** |  |  |  |  |
| Non-using^a^ | Ref | Ref | Ref |  |
| Occasional vaping / smoking | -0.01 (-0.12 to 0.09) | -0.00 (-0.11 to 0.10) | 0.92 |  |
| Daily vaping | **-0.11 (-0.20 to -0.01)** | 0.02 (-0.11 to 0.15) | 0.76 | Ref |
| Daily smoking | 0.12 (-0.03 to 0.27) | **0.21 (0.05 to 0.37)** | **0.01** | 0.19 (-0.01 to 0.39), p=0.075 |
| **3HPMA** |  |  |  |  |
| Non-using^a^ | Ref | Ref | Ref |  |
| Occasional vaping / smoking | 0.06 (-0.17 to 0.28) | 0.11 (-0.14 to 0.35) | 0.38 |  |
| Daily vaping | 0.05 (-0.16 to 0.26) | 0.23 (-0.08 to 0.53) | 0.14 | Ref |
| Daily smoking | **0.33 (0.00 to 0.66)** | **0.48 (0.10 to 0.86)** | **0.014** | 0.26 (-0.23 to 0.75), p=0.924 |
| **2CyEMA** |  |  |  |  |
| Non-using^a^ | Ref | Ref | Ref |  |
| Occasional vaping / smoking | **0.61 (0.27 to 0.94)** | **0.61 (0.26 to 0.97)** | **0.001** |  |
| Daily vaping | **0.44 (0.13 to 0.75)** | 0.41 (-0.03 to 0.86) | 0.07 | Ref |
| Daily smoking | **1.81 (1.32 to 2.29)** | **1.89 (1.33 to 2.45)** | **<0.001** | **1.48 (0.76 to 2.19), p<0.001** |
| **2HPMA** |  |  |  |  |
| Non-using^a^ | Ref | Ref | Ref |  |
| Occasional vaping / smoking | -0.09 (-0.26 to 0.08) | -0.03 (-0.21 to 0.16) | 0.80 |  |
| Daily vaping | -0.23 (-0.39 to -0.07) | -0.06 (-0.28 to 0.17) | 0.61 | Ref |
| Daily smoking | 0.11 (-0.15 to 0.36) | **0.29 (0.01 to 0.58)** | **0.043** | 0.35 (-0.01 to 0.72), p=0.066 |
| **1HMPeMA** |  |  |  |  |
| Non-using^a^ | Ref | Ref | Ref |  |
| Occasional vaping / smoking | 0.10 (-0.07 to 0.27) | 0.09 (-0.10 to 0.29) | 0.33 |  |
| Daily vaping | **0.22 (0.06 to 0.38)** | 0.22 (-0.02 to 0.46) | 0.072 | Ref |
| Daily smoking | **0.42 (0.17 to 0.67)** | **0.37 (0.07 to 0.67)** | **0.017** | 0.15 (-0.23 to 0.54), p=1 |
| **2HBeMA** |  |  |  |  |
| Non-using^a^ | Ref | Ref | Ref |  |
| Occasional vaping / smoking | 0.17 (-0.17 to 0.50) | 0.15 (-0.18 to 0.47) | 0.36 |  |
| Daily vaping | -0.20 (-0.51 to 0.12) | -0.08 (-0.48 to 0.33) | 0.71 | Ref |
| Daily smoking | 0.43 (-0.06 to 0.92) | 0.40 (-0.11 to 0.90) | 0.12 | 0.47 (-0.18 to 1.13), p=0.30 |
| **4HBeMA** |  |  |  |  |
| Non-using^a^ | Ref | Ref | Ref |  |
| Occasional vaping / smoking | 0.05 (-0.15 to 0.24) | 0.10 (-0.10 to 0.31) | 0.33 |  |
| Daily vaping | **-0.24 (-0.42 to -0.06)** | -0.08 (-0.33 to 0.18) | 0.54 | Ref |
| Daily smoking | **0.40 (0.11 to 0.68)** | **0.57 (0.25 to 0.89)** | **0.001** | **0.65 (0.24 to 1.06), p<0.001** |
| **3HMPMA** |  |  |  |  |
| Non-using^a^ | Ref | Ref | Ref |  |
| Occasional vaping / smoking | 0.06 (-0.07 to 0.19) | 0.10 (-0.04 to 0.24) | 0.16 |  |
| Daily vaping | -0.09 (-0.21 to 0.03) | 0.03 (-0.14 to 0.21) | 0.70 | Ref |
| Daily smoking | **0.20 (0.00 to 0.39)** | **0.33 (0.11 to 0.54)** | **0.004** | **0.29 (0.01 to 0.57), p=0.035** |
| **PhMA** |  |  |  |  |
| Non-using^a^ | Ref | Ref | Ref |  |
| Occasional vaping / smoking | -0.13 (-0.30 to 0.05) | -0.07 (-0.26 to 0.11) | 0.43 |  |
| Daily vaping | -0.05 (-0.22 to 0.11) | 0.11 (-0.13 to 0.34) | 0.36 | Ref |
| Daily smoking | 0.5 (-0.20 to 0.31) | 0.19 (-0.10 to 0.48) | 0.19 | 0.09 (-0.29 to 0.46), p=1 |

Abbreviations: FEV1 Forced Expired Volume in first second, FVC Forced Vital Capacity, FEV1/FVC modified Tiffeneau-Pinelli index, FEF 25-75 forced expiratory flow between 25% and 75% of forced vital capacity, LCI Lung Clerance Index LCI, FeNO Fraction of exhaled Nitric Oxide, 8-OHdG 8-Hydroxydesoxyguanosin. NNAL 4-(methylnitrosamino)-1-(3-pyridyl)-1-butanol, 1-OHP 1-Hydroxypyrene (a metabolite of pyrene), 1-Naphthol 1-Hydroxynaphthalene and 2-Naphthol 2-Hydroxynaphthalene (metabolites of naphthalene), CaHEMA Glycidamide mercapturic acid (a metabolite of acrylamide), 2HEMA 2-Hydroxyethylmercapturic acid (a metabolite of ethylene oxide, acrylonitrile, or vinyl chloride), 2CaEMA 2-Carbamoylethylmercapturic acid (a metabolite of acrylamide), 34HBMA Dihydroxybutylmercapturic acid (a metabolite of 1,3-butadiene), 3HPMA 3-Hydroxypropylmercapturic acid (a metabolite of acrolein), 2CyEMA 2-Cyanoethylmercapturic acid (a metabolite of acrylonitrile), 2HPMA 2-Hydroxypropylmercapturic acid (a metabolite of propylene oxide), 1HMPeMA, 2HBeMA , 4HBeMA Hydroxybutenyl mercapturic acids (metabolites of 1,3-butadiene, 4HBeMA specifically refers to 4-hydroxy-2-buten-1-yl-mercapturic acid), 3HMPMA 3-Hydroxy-1-methyl-propylmercapturic acid (a metabolite of crotonaldehyde), PhMA Phenylmercapturic acid (a metabolite of benzene). **Bold** if p<0.05 for higher exposures.

Missings: respiratory symptoms are missing in 2 participants (1 non-using, 1 occasional using adolescent), spirometry was missing in 1 participant from the daily vaping group. Urinary markers (oxidative stress, PAH, VOC) are missing in 13 participants (10 non-using, 3 occasional using adolescents). No covariables were missing.

a Non-using participants reported no vaping or smoking in the past 12 months or never vaping / never smoking but might be exposed to second-hand smoke.

b Models were adjusted for sex, age, education, exposure to second-hand smoke, oven / chimney at home, heavy traffic near place of living, any atopic disease.

c p-value come from a t-test for linear regressions and from a Wald test for logistic regressions, comparing occasional vaping/smoking, daily vaping or daily smoking to non-using adolescents (reference group).

d Contrast estimates, corrected 95% confidence interval and p-value comes from a pairwise comparisons of marginal linear predictions, applying Bonferroni correction, comparing daily smoking and daily vaping adolescents.

e FeNO was log transformed as its distribution is right skewed.

f Values (ng/ml) in urine normalized to creatinine concentration (mg/ml) in urine (results in ng/mg creatinine). Urinary analyses of oxidative stress biomarkers, and the metabolites of PAH and VOC were available for 59 participants (19 (66%) non-using, 15 (83%) occasional users, 20 (100%) daily vaping, 5 (100%) daily smoking). All PAH and VOC metabolites were log10 transformed to achieve normal distribution.

# Appendix Table 3: Demographics of the participants with **self-reported** exposure categories.

| Variable | All participants  (N=89) | Non-using^a^  (n=35) | Occasional vaping / smoking  (n=21) | Daily vaping  (n=22) | Daily smoking (n=11) |
| --- | --- | --- | --- | --- | --- |
| Age years, median (Q1; Q3) | 17 (16; 18) | 17 (16; 17) | 17 (16; 17) | 19 (18; 20) | 18 (17; 20) |
| Sex, women N (%) | 45 (51%) | 16 (46%) | 9 (43%) | 16 (73%) | 4 (36%) |
| Education: secondary or tertiary (academic), N (%) | 49 (55%) | 28 (80%) | 9 (43%) | 10 (45%) | 2 (18%) |
| Exposure to any second-hand smoke, N (%) | 33 (37%) | 3 (9%) | 10 (48%) | 13 (59%) | 7 (64%) |
| Oven or fireplace at home, N (%)^b^ | 36 (40%) | 14 (40%) | 11 (52%) | 6 (27%) | 5 (45%) |
| Trucks driving through street where participant lives, often or very often, N (%) | 23 (26%) | 8 (23%) | 4 (19%) | 8 (36%) | 3 (27%) |
| Self-reported atopic disease: Asthma, N (%) | 28 (31%) | 13 (37%) | 6 (29%) | 6 (27%) | 3 (27%) |
| BMI, kg/m^2^, median (Q1; Q3) | 21.5 (19.3; 23.6) | 20.0 (18.6; 21.6) | 22.2 (20.5; 24.0) | 25.3 (21.1; 27.8) | 21.5 (19.5; 23.0) |
| Atopic dermatitis, N (%) | 4 (4%) | 1 (3%) | 2 (10%) | 1 (5%) | 0 (0%) |
| Asthma, N (%) | 10 (11%) | 4 (11%) | 3 (14%) | 1 (5%) | 2 (18%) |
| Number of members of household also smoking, median (Q1; Q3) | 0 (0; 1) | 0 (0; 0) | 0 (0; 1) | 0 (0; 1) | 1 (1; 1) |
| Quartiles of friends or family members smoking, median (Q1; Q3) ^c^ | 1 (1; 3) | 1 (0; 1) | 1 (1; 3) | 3 (1; 3) | 3 (2; 4) |
| Quartiles of friends or family members vaping, median (Q1; Q3) ^c^ | 1 (0; 2) | 0 (0; 1) | 1 (0; 1) | 2 (1; 3) | 2 (1; 3) |

Abbreviations: Q1 quartile 1, Q3 quartile 3

a Abstainers reported no vaping or smoking in the past 12 months or never vaping / never smoking

b Living close to a busy road

c Quartiles refer to the following proportions: (0) 0%; (1) 1-25%; (2) 26-50%; (3) 51-75%; (4) 76-100%

# Appendix Table 4: Unadjusted and adjusted models on **self-reported** exposure categories. Linear regression models for continuous outcomes (providing b-estimates), logistic regression models for binary outcomes (providing Odds Ratio OR).

| **Respiratory health measures** | **Unadjusted, b-coefficient / OR and 95 % CI** | **Fully adjusted, b-coefficient / OR and 95 % CI^b^** | **Fully adjusted p-value^c^** | **Comparing daily vaping and smoking (95% CI) and p-value^d^** |
| --- | --- | --- | --- | --- |
| **RESPIRATORY SYMPTOMS** |  |  |  |  |
| **Longest episode without coughing in days** | **b-coefficient** | **b-coefficient** |  |  |
| Non-using^a^ | Ref | Ref | Ref |  |
| Occasional vaping / smoking | -19 (-77 to 38) | -25 (-87 to 36) | 0.42 |  |
| Daily vaping | -106 (-161 to -51) | **-118 (-193 to -42)** | **0.003** | Ref |
| Daily smoking | -125 (-195 to -56) | **-130 (-214 to -47)** | **0.003** | -13 (-119 to 94), p=1.0 |
| **Ever wheezing in the past 12 months (yes/no)** | **Odds Ratio** | **Odds Ratio** |  |  |
| Non-using^a^ | Ref | Ref | Ref |  |
| Occasional vaping / smoking | n/a | n/a | n/a |  |
| Daily vaping | 1.1 (0.2 to 5.7) | 4.0 (0.4 to 40.2) | 0.24 | Ref |
| Daily smoking | 6.0 (1.2 to 29.4) | **13.7 (1.3 to 146.1)** | **0.030** | 1.2 (-1.7 to 4.2), p=1.0 |
| **Number of episodes of cold in the past 12 months** | **b-coefficient** | **b-coefficient** |  |  |
| Non-using^a^ | Ref | Ref | Ref |  |
| Occasional vaping / smoking | 0 (-1 to 1) | 0 (-1 to 2) | 0.88 |  |
| Daily vaping | 2 (1 to 3) | **3 (1 to 5)** | **0.001** | Ref |
| Daily smoking | 2 (0 to 4) | **2 (1 to 4)** | **0.014** | -0 (-3 to 2), p=1.0 |
| **Dry coughing at night in the past 12 months (yes/no)** | **Odds Ratio** | **Odds Ratio** |  |  |
| Non-using^a^ | Ref | Ref | Ref |  |
| Occasional vaping / smoking | 4.1 (0.7 to 25.2) | 4.6 (0.6 to 33.8) | 0.14 |  |
| Daily vaping | 12.9 (2.5 to 67.8) | **40.2 (3.9 to 417.4)** | **0.002** | Ref |
| Daily smoking | 12.9 (2.0 to 82.8) | **22.4 (2.4 to 211.9)** | **0.007** | -0.6 (-3.1 to 1.9), p=1.0 |
| **Coughing during sports in the past 12 months (yes/no)** | **Odds Ratio** | **Odds Ratio** |  |  |
| Non-using^a^ | Ref | Ref | Ref |  |
| Occasional vaping / smoking | 2.6 (0.7 to 9.2) | 2.4 (0.6 to 9.4) | 0.22 |  |
| Daily vaping | 3.3 (1.0 to 10.8) | **7.9 (1.3 to 48.9)** | **0.026** | Ref |
| Daily smoking | 5.8 (1.3 to 25.6) | **9.1 (1.33 to 61.9)** | **0.024** | 0.1 (-2.2 to 2.5), p=1.0 |
| **Mucus expectoration without a cold in the past 12 months (yes/no)** | **Odds Ratio** | **Odds Ratio** |  |  |
| Non-using^a^ | Ref | Ref | Ref |  |
| Occasional vaping / smoking | 2.4 (0.7 to 7.8) | 2.4 (0.6 to 8.8) | 0.19 |  |
| Daily vaping | 7.1 (2.1 to 23.9) | **6.3 (1.1 to 34.5)** | **0.034** | Ref |
| Daily smoking | 7.1 (1.5 to 32.9) | **6.3 (1.0 to 38.5)** | **0.046** | -0.0 (-2.4 to 2.4), p=1.0 |
| **LUNG FUNCTION MEASURES** | **b-coefficient** | **b-coefficient** |  |  |
| **FEV1 z-score** |  |  |  |  |
| Non-using^a^ | Ref | Ref | Ref |  |
| Occasional vaping / smoking | 0.4 (-0.1 to 1.0) | 0.5 (-0.1 to 1.0) | 0.13 |  |
| Daily vaping | -0.1 (-0.7 to 0.4) | 0.1 (-0.7 to 0.9) | 0.83 | Ref |
| Daily smoking | 0.2 (-0.5 to 0.9) | 0.4 (-0.5 to 1.3) | 0.38 | 0.3 (-0.8 to 1.4), p=1 |
| **FVC z-score** |  |  |  |  |
| Non-using^a^ | Ref | Ref | Ref |  |
| Occasional vaping / smoking | 0.3 (-0.2 to 0.8) | 0.5 (-0.1 to 1.0) | 0.089 |  |
| Daily vaping | 0.1 (-0.3 to 0.6) | 0.3 (-0.4 to 1.0) | 0.40 | Ref |
| Daily smoking | -0.0 (-0.7 to 0.6) | 0.3 (-0.5 to 1.1) | 0.45 | -0.0 (-1.0 to 1.0), p=1 |
| **FEV1/FVC z-score** |  |  |  |  |
| Non-using^a^ | Ref | Ref | Ref |  |
| Occasional vaping / smoking | 0.1 (-1.0 to 1.1) | -0.1 (-1.3 to 1.0) | 0.84 |  |
| Daily vaping | -0.3 (-1.4 to 0.7) | -0.5 (-2.0 to 1.1) | 0.55 | Ref |
| Daily smoking | 0.3 (-1.0 to 1.6) | 0.0 (-1.6 to 1.7) | 0.96 | 0.5 (-1.6 to 2.6), p=1 |
| **FEF 25-75 z-score** |  |  |  |  |
| Non-using^a^ | Ref | Ref | Ref |  |
| Occasional vaping / smoking | -0.3 (-0.9 to 0.2) | -0.4 (-1.0 to 0.2) | 0.19 |  |
| Daily vaping | -0.1 (-0.6 to 0.5) | -0.0 (-0.8 to 0.7) | 0.93 | Ref |
| Daily smoking | 0.0 (-0.6 to 0.7) | 0.1 (-0.8 to 0.9) | 0.90 | 0.1 (-0.9 to 1.2), p=1 |
| **FRC z-score** |  |  |  |  |
| Non-using^a^ | Ref | Ref | Ref |  |
| Occasional vaping / smoking | 0.3 (-0.2 to 0.8) | 0.4 (-0.1 to 0.9) | 0.12 |  |
| Daily vaping | -0.3 (-0.8 to 0.1) | 0.0 (-0.6 to 0.7) | 0.94 | Ref |
| Daily smoking | 0.1 (-0.5 to 0.7) | 0.2 (-0.5 to 0.9) | 0.56 | 0.2 (-0.7 to 1.1), p=1 |
| **TLC z-score** |  |  |  |  |
| Non-using^a^ | Ref | Ref | Ref |  |
| Occasional vaping / smoking | 0.4 (-0.1 to 0.8) | 0.4 (-0.1 to 0.9) | 0.10 |  |
| Daily vaping | -0.0 (-0.5 to 0.4) | 0.0 (-0.6 to 0.6) | 0.92 | Ref |
| Daily smoking | 0.0 (-0.5 to 0.6) | 0.0 (-0.6 to 0.7) | 0.89 | 0.0 (-0.8 to 0.9), p=1 |
| **Raw** |  |  |  |  |
| Non-using^a^ | Ref | Ref | Ref |  |
| Occasional vaping / smoking | -0.02 (-0.06 to 0.01) | -0.01 (-0.05 to 0.02) | 0.44 |  |
| Daily vaping | -0.01 (-0.05 to 0.02) | 0.01 (-0.04 to 0.05) | 0.81 | Ref |
| Daily smoking | -0.03 (-0.07 to 0.01) | -0.01 (-0.06 to 0.04) | 0.78 | - 0.01 (-0.07 to 0.05), p=1 |
| **LCI** |  |  |  |  |
| Non-using^a^ | Ref | Ref | Ref |  |
| Occasional vaping / smoking | -0.2 (-0.5 to -0.0) | **-0.3 (-0.5 to -0.0)** | **0.032** |  |
| Daily vaping | -0.1 (-0.4 to 0.1) | -0.1 (-0.4 to 0.2) | 0.43 | Ref |
| Daily smoking | -0.0 (-0.3 to 0.3) | -0.1 (-0.5 to 0.2) | 0.52 | 0.0 (-0.4 to 0.4), p=1 |
| **Log FeNO^e^** |  |  |  |  |
| Non-using^a^ | Ref | Ref | Ref |  |
| Occasional vaping / smoking | -0.0 (-0.4 to 0.4) | 0.0 (-0.4 to 0.4) | 0.84 |  |
| Daily vaping | 0.1 (-0.3 to 0.4) | 0.2 (-0.3 to 0.7) | 0.46 | Ref |
| Daily smoking | 0.0 (-0.4 to 0.5) | 0.1 (-0.4 to 0.7) | 0.63 | -0.1 (-0.8 to 0.7), p=1 |
| **OXIDATIVE STRESS METABOLITES, ng/mg creatinine^f^** | **b-coefficient** | **b-coefficient** |  |  |
| **8-OHdG** |  |  |  |  |
| Non-using^a^ | Ref | Ref | Ref |  |
| Occasional vaping / smoking | 0.3 (-0.7 to 1.3) | 0.4 (-0.6 to 1.4) | 0.46 |  |
| Daily vaping | -0.1 (-1.0 to 0.8) | 0.0 (-1.1 to 1.2) | 0.97 | Ref |
| Daily smoking | 0.8 (-0.3 to 1.9) | 0.8 (-0.5 to 2.1) | 0.21 | 0.8 (-0.7 to 2.4), p=0.95 |
| **8- Isoprostane** |  |  |  |  |
| Non-using^a^ | Ref | Ref | Ref |  |
| Occasional vaping / smoking | 0.00 (-0.06 to 0.06) | 0.00 (-0.06 to 0.06) | 0.97 |  |
| Daily vaping | -0.02 (-0.07 to 0.03) | -0.02 (-0.09 to 0.06) | 0.66 | Ref |
| Daily smoking | 0.03 (-0.03 to 0.10) | 0.04 (-0.05 to 0.12) | 0.38 | 0.05 (-0.05 to 0.15), p=0.89 |
| **PAH / VOC METABOLITES, ng/mg creatinine^f^** | **b-coefficient** | **b-coefficient** |  |  |
| **1-OHP** |  |  |  |  |
| Non-using^a^ | Ref | Ref | Ref |  |
| Occasional vaping / smoking | 0.01 (-0.23 to 0.24) | -0.00 (-0.24 to 0.24) | 0.99 |  |
| Daily vaping | 0.16 (-0.05 to 0.37) | 0.02 (-0.26 to 0.30) | 0.88 | Ref |
| Daily smoking | **0.40 (0.14 to 0.67)** | **0.33 (0.01 to 0.65)** | **0.043** | 0.31 (-0.07 to 0.69), p=0.18 |
| **1-Naphthol** |  |  |  |  |
| Non-using^a^ | Ref | Ref | Ref |  |
| Occasional vaping / smoking | 0.21 (-0.18 to 0.60) | 0.26 (-0.16 to 0.68) | 0.22 |  |
| Daily vaping | -0.15 (-0.51 to 0.21) | -0.07 (-0.56 to 0.41) | 0.76 | Ref |
| Daily smoking | **0.54 (0.10 to 0.99)** | **0.67 (0.11 to 1.22)** | **0.019** | **0.74 (0.83 to 1.40), p=0.019** |
| **2-Naphthol** |  |  |  |  |
| Non-using^a^ | Ref | Ref | Ref |  |
| Occasional vaping / smoking | 0.07 (-0.16 to 0.30) | 0.13 (-0.11 to 0.36) | 0.27 |  |
| Daily vaping | -0.01 (-0.22 to 0.20) | -0.03 (-0.30 to 0.25) | 0.85 | Ref |
| Daily smoking | 0.08 (-0.18 to 0.35) | 0.17 (-0.14 to 0.48) | 0.26 | 0.20 (-0.17 to 0.57), p=0.86 |
| **CaHEMA** |  |  |  |  |
| Non-using^a^ | Ref | Ref | Ref |  |
| Occasional vaping / smoking | -0.02 (-0.23 to 0.18) | 0.03 (-0.18 to 0.24) | 0.78 |  |
| Daily vaping | **0.27 (0.08 to 0.46)** | 0.15 (-0.09 to 0.39) | 0.23 | Ref |
| Daily smoking | **0.40 (0.16 to 0.63)** | **0.37 (0.09 to 0.64)** | **0.010** | 0.22 (-0.11 to 0.55), p=0.44 |
| **2HEMA** |  |  |  |  |
| Non-using^a^ | Ref | Ref | Ref |  |
| Occasional vaping / smoking | -0.06 (-0.27 to 0.16) | -0.02 (-0.25 to 0.21) | 0.86 |  |
| Daily vaping | **-0.20 (-0.39 to -0.00)** | -0.19 (-0.46 to 0.08) | 0.16 | Ref |
| Daily smoking | -0.03 (-0.28 to 0.21) | 0.02 (-0.28 to 0.33) | 0.88 | 0.21 (-0.15 to 0.58), p=0.69 |
| **2CaEMA** |  |  |  |  |
| Non-using^a^ | Ref | Ref | Ref |  |
| Occasional vaping / smoking | 0.01 (-0.22 to 0.24) | 0.06 (-0.18 to 0.31) | 0.60 |  |
| Daily vaping | -0.05 (-0.27 to 0.16) | -0.07 (-0.35 to 0.21) | 0.62 | Ref |
| Daily smoking | **0.28 (0.01 to 0.54)** | **0.33 (0.01 to 0.65)** | **0.044** | **0.40 (0.20 to 0.78), p=0.034** |
| **34HBMA** |  |  |  |  |
| Non-using^a^ | Ref | Ref | Ref |  |
| Occasional vaping / smoking | -0.02 (-0.12 to 0.09) | -0.00 (-0.12 to 0.11) | 0.93 |  |
| Daily vaping | **-0.12 (-0.21 to -0.02)** | -0.04 (-0.16 to 0.09) | 0.57 | Ref |
| Daily smoking | 0.01 (-0.11 to 0.13) | 0.09 (-0.06 to 0.23) | 0.24 | 0.12 (-0.50 to 0.30), p=0.34 |
| **3HPMA** |  |  |  |  |
| Non-using^a^ | Ref | Ref | Ref |  |
| Occasional vaping / smoking | 0.06 (-0.15 to 0.28) | 0.11 (-0.12 to 0.34) | 0.35 |  |
| Daily vaping | 0.08 (-0.12 to 0.27) | 0.17 (-0.10 to 0.43) | 0.21 | Ref |
| Daily smoking | **0.25 (0.00 to 0.50)** | **0.34 (0.03 to 0.64)** | **0.031** | 0.17 (-0.19 to 0.53), p=1.00 |
| **2CyEMA** |  |  |  |  |
| Non-using^a^ | Ref | Ref | Ref |  |
| Occasional vaping / smoking | **0.60 (0.26 to 0.94)** | **0.61 (0.25 to 0.98)** | **0.001** |  |
| Daily vaping | **0.42 (0.11 to 0.74)** | 0.27 (-0.15 to 0.69) | 0.20 | Ref |
| Daily smoking | **1.46 (1.07 to 1.86)** | **1.37 (0.89 to 1.85)** | **<0.001** | **1.10 (0.53 to 1.67), p<0.001** |
| **2HPMA** |  |  |  |  |
| Non-using^a^ | Ref | Ref | Ref |  |
| Occasional vaping / smoking | -0.08 (-0.24 to 0.09) | -0.02 (-0.20 to 0.16) | 0.80 |  |
| Daily vaping | **-0.24 (-0.39 to -0.08)** | -0.15 (-0.36 to 0.06) | 0.15 | Ref |
| Daily smoking | -0.06 (-0.26 to 0.13) | 0.06 (-0.18 to 0.30) | 0.63 | 0.21 (-0.73 to 0.49), p=0.29 |
| **1HMPeMA** |  |  |  |  |
| Non-using^a^ | Ref | Ref | Ref |  |
| Occasional vaping / smoking | 0.15 (-0.4 to 0.33) | 0.11 (-0.09 to 0.30) | 0.28 |  |
| Daily vaping | **0.24 (0.07 to 0.41)** | 0.16 (-0.06 to 0.38) | 0.15 | Ref |
| Daily smoking | **0.31 (0.10 to 0.53)** | 0.21 (-0.05 to 0.46) | 0.11 | 0.05 (-0.26 to 0.35), p=1 |
| **2HBeMA** |  |  |  |  |
| Non-using^a^ | Ref | Ref | Ref |  |
| Occasional vaping / smoking | 0.21 (-0.14 to 0.56) | 0.19 (-0.17 to 0.54) | 0.30 |  |
| Daily vaping | -0.21 (-0.53 to 0.12) | -0.22 (-0.62 to 0.19) | 0.29 | Ref |
| Daily smoking | 0.17 (-0.24 to 0.57) | 0.17 (-0.29 to 0.64) | 0.46 | 0.39 (-0.16 to 0.94), p=0.36 |
| **4HBeMA** |  |  |  |  |
| Non-using^a^ | Ref | Ref | Ref |  |
| Occasional vaping / smoking | 0.05 (-0.17 to 0.27) | 0.09 (-0.14 to 0.32) | 0.46 |  |
| Daily vaping | **-0.23 (-0.43 to -0.03)** | -0.17 (-0.44 to 0.09) | 0.19 | Ref |
| Daily smoking | 0.15 (-0.10 to 0.40) | 0.19 (-0.11 to 0.49) | 0.21 | **0.36 (0.01 to 0.72), p=0.045** |
| **3HMPMA** |  |  |  |  |
| Non-using^a^ | Ref | Ref | Ref |  |
| Occasional vaping / smoking | 0.08 (-0.06 to 0.22) | 0.11 (-0.04 to 0.25) | 0.14 |  |
| Daily vaping | -0.08 (-0.20 to 0.05) | -0.05 (-0.22 to 0.11) | 0.54 | Ref |
| Daily smoking | 0.14 (-0.02 to 0.03) | 0.17 (-0.02 to 0.36) | 0.078 | 0.22 (-0.04 to 0.45), p=0.057 |
| **PhMA** |  |  |  |  |
| Non-using^a^ | Ref | Ref | Ref |  |
| Occasional vaping / smoking | -0.10 (-0.28 to 0.07) | -0.06 (-0.24 to 0.13) | 0.55 |  |
| Daily vaping | -0.05 (-0.21 to 0.11) | 0.09 (-0.12 to 0.30) | 0.38 | Ref |
| Daily smoking | -0.06 (-0.25 to 0.14) | 0.09 (-0.15 to 0.33) | 0.44 | -0.00 (-0.29 to 0.29), p=1 |

Abbreviations: FEV1 Forced Expired Volume in first second, FVC Forced Vital Capacity, FEV1/FVC modified Tiffeneau-Pinelli index, FEF 25-75 forced expiratory flow between 25% and 75% of forced vital capacity, LCI Lung Clerance Index LCI, FeNO Fraction of exhaled Nitric Oxide, OHdG 8-Hydroxydesoxyguanosin. NNAL 4-(methylnitrosamino)-1-(3-pyridyl)-1-butanol, 1-OHP 1-Hydroxypyrene (a metabolite of pyrene), 1-Naphthol 1-Hydroxynaphthalene and 2-Naphthol 2-Hydroxynaphthalene (metabolites of naphthalene), CaHEMA Glycidamide mercapturic acid (a metabolite of acrylamide), 2HEMA 2-Hydroxyethylmercapturic acid (a metabolite of ethylene oxide, acrylonitrile, or vinyl chloride), 2CaEMA 2-Carbamoylethylmercapturic acid (a metabolite of acrylamide), 34HBMA Dihydroxybutylmercapturic acid (a metabolite of 1,3-butadiene), 3HPMA 3-Hydroxypropylmercapturic acid (a metabolite of acrolein), 2CyEMA 2-Cyanoethylmercapturic acid (a metabolite of acrylonitrile), 2HPMA 2-Hydroxypropylmercapturic acid (a metabolite of propylene oxide), 1HMPeMA, 2HBeMA , 4HBeMA Monohydroxybutenyl-mercapturic acids (metabolites of 1,3-butadiene, 4HBeMA specifically refers to 4-hydroxy-2-buten-1-yl-mercapturic acid), 3HMPMA 3-Hydroxy-1-methyl-propylmercapturic acid (a metabolite of crotonaldehyde), PhMA Phenylmercapturic acid (a metabolite of benzene). **Bold** if p<0.05 for higher exposures.

Missings: respiratory symptoms are missing in 4 participants (2 non-using, 2 occasional using adolescent), spirometry was missing in 1 participant from the daily vaping group. Urinary markers (oxidative stress, PAH, VOC metabolites) are missing in 21 participants (15 non-using, 5 occasional using, 1 daily smoking adolescents). No covariables were missing.

a Non-using participants reported no vaping or smoking in the past 12 months or never vaping / never smoking but might be exposed to second-hand smoke.

b Models were adjusted for sex, age, education, exposure to second-hand smoke, oven / chimney at home, heavy traffic near place of living, any atopic disease.

c p-value come from a t-test, comparing occasional vaping/smoking, daily vaping or daily smoking to non-using adolescents (reference group).

d Contrast estimates, corrected 95% confidence interval and p-value comes from a pairwise comparisons of marginal linear predictions, applying Bonferroni correction, comparing daily smoking and daily vaping adolescents.

e FeNO was log transformed as its distribution is right skewed.

f Values (ng/ml) in urine normalized to creatinine concentration (mg/ml) in urine (results in ng/mg creatinine). All PAH and VOC metabolites were log10 transformed to achieve normal distribution.

# Appendix Table 5: Demographics of the participants using **lab-based** exposure categories

| Variable | All participants  (N=81) | Nicotine Abstaining^a^  (n=41) | Non-tobacco nicotine use  (n=32) | Active smoking (n=8) |
| --- | --- | --- | --- | --- |
| Age years, median (Q1; Q3) | 17 (16; 19) | 17 (16; 17) | 19 (17; 20) | 17 (16; 20) |
| Sex, women N (%) | 41 (51%) | 19 (46%) | 19 (59%) | 3 (38%) |
| Education: secondary or tertiary (academic), N (%) | 41 (51%) | 28 (68%) | 12 (38%) | 1 (12%) |
| Exposure to any second-hand smoke, N (%) | 32 (40%) | 9 (22%) | 18 (56%) | 5 (62%) |
| Oven or fireplace at home, N (%)^b^ | 33 (41%) | 17 (41%) | 12 (38%) | 4 (50%) |
| Trucks driving through street where participant lives, often or very often, N (%) | 22 (27%) | 10 (24%) | 10 (31%) | 2 (25%) |
| Self-reported atopic disease: Asthma, N (%) | 24 (30%) | 13 (32%) | 8 (25%) | 3 (38%) |
| BMI, kg/m^2^, median (Q1; Q3) | 21.5 (19.3; 24.3) | 20.1 (18.7; 22.1) | 23.1 (20.1; 26.7) | 21.9 (20.3; 24.5) |
| Atopic dermatitis, N (%) | 4 (5%) | 3 (7%) | 1 (3%) | 0 (0%) |
| Asthma, N (%) | 9 (11%) | 6 (15%) | 2 (6%) | 1 (12%) |
| Number of members of household also smoking, median (Q1; Q3) | 0 (0; 1) | 0 (0; 0) | 0 (0; 1) | 1 (1; 2) |
| Quartiles of friends or family members smoking, median (Q1; Q3) ^c^ | 1 (1; 3) | 1 (1; 1) | 2 (1; 3) | 3 (2; 4) |
| Quartiles of friends or family members vaping, median (Q1; Q3) ^c^ | 1 (0; 2) | 0 (0; 1) | 2 (1; 3) | 2 (0; 3) |

Abbreviations: Q1 quartile 1, Q3 quartile 3

a Nicotine abstaining participants had urinary cotinine and NNAL levels below cut-off

b Living close to a busy road

c Quartiles refer to the following proportions: (0) 0%; (1) 1-25%; (2) 26-50%; (3) 51-75%; (4) 76-100%

# Appendix Table 6: Unadjusted and adjusted models with **lab-based** exposure categories. Linear regression models for continuous outcomes (providing b-estimates), logistic regression models for binary outcomes (providing Odds Ratio OR).

| **Respiratory health measures** | **Unadjusted, b-coefficient / OR and 95 % CI** | **Fully adjusted, b-coefficient / OR and 95 % CI^b^** | **Fully adjusted p-value^c^** | **Comparing daily vaping and smoking (95% CI) and p-value^d^** |
| --- | --- | --- | --- | --- |
| **RESPIRATORY SYMPTOMS** |  |  |  |  |
| **Longest episode without coughing in days** | **b-coefficient** | **b-coefficient** |  |  |
| Nicotine Abstaining^a^ | Ref | Ref | Ref |  |
| Non-tobacco nicotine use | -96 (-146 to -46) | **-97 (-166 to -28)** | **0.006** | Ref |
| Active smoking | -107 (-188 to -26) | **-118 (-206 to -29)** | **0.010** | -20 (-127 to 86), p=1.0 |
| **Ever wheezing in the past 12 months (yes/no)** | **Odds Ratio** | **Odds Ratio** |  |  |
| Nicotine Abstaining^a^ | Ref | Ref | Ref |  |
| Non-tobacco nicotine use | 0.7 (0.2 to 3.2) | 0.4 (0.0 to 5.5) | 0.53 | Ref |
| Active smoking | 6.8 (1.3 to 36.3) | 11.5 (1.0 to 136.4) | 0.053 | **3.2 (0.1 to 6.4), p=0.043** |
| **Number of episodes of cold in the past 12 months** | **b-coefficient** | **b-coefficient** |  |  |
| Nicotine Abstaining^a^ | Ref | Ref | Ref |  |
| Non-tobacco nicotine use | 1 (0 to 2) | 1 (-0 to 3) | 0.20 | Ref |
| Active smoking | 2 (0 to 3) | 1 (-1 to 3) | 0.20 | 0 (-2 to 3), p=1 |
| **Dry coughing at night in the past 12 months (yes/no)** | **Odds Ratio** | **Odds Ratio** |  |  |
| Nicotine Abstaining^a^ | Ref | Ref | Ref |  |
| Non-tobacco nicotine use | 3.6 (1.1 to 11.7) | 3.1 (0.6 to 17.0) | 0.20 | Ref |
| Active smoking | 6.8 (1.3 to 36.3) | 6.5 (0.9 to 48.5) | 0.070 | 0.7 (-1.5 to 2.9), p=1 |
| **Coughing during sports in the past 12 months (yes/no)** | **Odds Ratio** | **Odds Ratio** |  |  |
| Nicotine Abstaining^a^ | Ref | Ref | Ref |  |
| Non-tobacco nicotine use | 2.2 (0.8 to 6.1) | 4.4 (1.0 to 19.7) | 0.053 | Ref |
| Active smoking | 18.9 (2.1 to 173.6) | **27.3 (2.3 to 317.5)** | **0.008** | 1.8 (-1.0 to 4.7), p=1 |
| **Mucus expectoration without a cold in the past 12 months (yes/no)** | **Odds Ratio** | **Odds Ratio** |  |  |
| Nicotine Abstaining^a^ | Ref | Ref | Ref |  |
| Non-tobacco nicotine use | 2.9 (1.1 to 7.7) | 1.6 (0.4 to 6.4) | 0.52 | Ref |
| Active smoking | 14.0 (1.6 to 126.2) | **11.1 (1.1 to 115.4)** | **0.043** | 1.9 (-0.9 to 4.8), p=0.32 |
| **LUNG FUNCTION MEASURES** | **b-coefficient** | **b-coefficient** |  |  |
| **FEV1 z-score** |  |  |  |  |
| Nicotine Abstaining^a^ | Ref | Ref | Ref |  |
| Non-tobacco nicotine use | -0.0 (-0.5 to 0.5) | 0.1 (-0.6 to 0.8) | 0.73 | Ref |
| Active smoking | 0.1 (-0.7 to 0.9) | 0.1 (-0.7 to 1.0) | 0.81 | -0.0 (-1.1 to 1.1), p=1 |
| **FVC z-score** |  |  |  |  |
| Nicotine Abstaining^a^ | Ref | Ref | Ref |  |
| Non-tobacco nicotine use | 0.1 (-0.3 to 0.5) | 0.4 (-0.2 to 1.0) | 0.19 | Ref |
| Active smoking | 0.1 (-0.6 to 0.8) | 0.4 (-0.4 to 1.2) | 0.29 | 0.0 (-0.9 to 1.0), p=1 |
| **FEV1/FVC z-score** |  |  |  |  |
| Nicotine Abstaining^a^ | Ref | Ref | Ref |  |
| Non-tobacco nicotine use | -0.2 (-1.2 to 0.7) | -0.6 (-1.9 to 0.7) | 0.37 | Ref |
| Active smoking | -0.2 (-1.7 to 1.3) | -0.6 (-2.3 to 1.0) | 0.46 | -0.0 (-2.1 to 2.0), p=1 |
| **FEF 25-75 z-score** |  |  |  |  |
| Nicotine Abstaining^a^ | Ref | Ref | Ref |  |
| Non-tobacco nicotine use | -0.2 (-0.8 to 0.4) | 0.0 (-0.9 to 0.9) | 0.99 | Ref |
| Active smoking | 0.1 (-1.1 to 0.8) | -0.2 (-1.4 to 0.9) | 0.69 | -0.2 (-1.8 to 1.3), p=1 |
| **FRC z-score** |  |  |  |  |
| Nicotine Abstaining^a^ | Ref | Ref | Ref |  |
| Non-tobacco nicotine use | -0.2 (-0.6 to 0.3) | 0.1 (-0.5 to 0.7) | 0.75 | Ref |
| Active smoking | 0.1 (-0.6 to 0.8) | 0.2 (-0.6 to 0.9) | 0.68 | 0.1 (-0.8 to 1.0), p=1 |
| **TLC z-score** |  |  |  |  |
| Nicotine Abstaining^a^ | Ref | Ref | Ref |  |
| Non-tobacco nicotine use | 0.0 (-0.4 to 0.4) | 0.1 (-0.4 to 0.7) | 0.61 | Ref |
| Active smoking | -0.1 (-0.7 to 0.6) | -0.0 (-0.7 to 0.7) | 0.94 | -0.2 (-1.0 to 0.7), p=1 |
| **Raw** |  |  |  |  |
| Nicotine Abstaining^a^ | Ref | Ref | Ref |  |
| Non-tobacco nicotine use | -0.01 (-0.04 to 0.02) | 0.01 (-0.03 to 0.05) | 0.69 | Ref |
| Active smoking | -0.04 (-0.09 to 0.01) | -0.02 (-0.07 to 0.03) | 0.37 | -0.03 (-0.09 to 0.03), p=0.67 |
| **LCI** |  |  |  |  |
| Nicotine Abstaining^a^ | Ref | Ref | Ref |  |
| Non-tobacco nicotine use | -0.1 (-0.3 to 0.1) | -0.2 (-0.4 to 0.1) | 0.29 | Ref |
| Active smoking | -0.2 (-0.5 to 0.2) | -0.2 (-0.6 to 0.2) | 0.26 | -0.1 (-0.5 to 0.4), p=1 |
| **Log FeNO^e^** |  |  |  |  |
| Nicotine Abstaining^a^ | Ref | Ref | Ref |  |
| Non-tobacco nicotine use | -0.0 (-0.3 to 0.3) | -0.1 (-0.5 to 0.4) | 0.81 | Ref |
| Active smoking | 0.1 (-0.4 to 0.6) | 0.1 (-0.4 to 0.7) | 0.66 | 0.2 (-0.5 to 0.8), p=1 |
| **OXIDATIVE STRESS METABOLITES, ng/mg creatinine^f^** | **b-coefficient** | **b-coefficient** |  |  |
| **OHdG** |  |  |  |  |
| Nicotine Abstaining^a^ | Ref | Ref | Ref |  |
| Non-tobacco nicotine use | -0.1 (-0.9 to 0.6) | 0.1 (-0.9 to 1.0) | 0.91 | Ref |
| Active smoking | 0.9 (-0.2 to 2.1) | **1.2 (0.0 to 2.4)** | **0.044** | 1.2 (-0.2 to 2.6), p=0.13 |
| **8- Isoprostane** |  |  |  |  |
| Nicotine Abstaining^a^ | Ref | Ref | Ref |  |
| Non-tobacco nicotine use | 0.00 (-0.04 to 0.04) | 0.01 (-0.05 to 0.07) | 0.63 | Ref |
| Active smoking | 0.01 (-0.0.6 to 0.08) | 0.02 (-0.06 to 0.09) | 0.68 | 0.00 (-0.09 to 0.09), p=1 |
| **PAH / VOC METABOLITES, ng/mg creatinine^f^** | **b-coefficient** | **b-coefficient** |  |  |
| **1-OHP** |  |  |  |  |
| Nicotine Abstaining^a^ | Ref | Ref | Ref |  |
| Non-tobacco nicotine use | **0.20 (0.03 to 0.38)** | 0.08 (-0.15 to 0.31) | 0.50 | Ref |
| Active smoking | **0.40 (0.12 to 0.68)** | **0.41 (0.11 to 0.70)** | **0.008** | 0.33 (-0.02 to 0.68), p=0.071 |
| **1-Naphthol** |  |  |  |  |
| Nicotine Abstaining^a^ | Ref | Ref | Ref |  |
| Non-tobacco nicotine use | 0.04 (-0.25 to 0.33) | 0.16 (-0.23 to 0.55) | 0.41 | Ref |
| Active smoking | **0.81 (0.35 to 1.26)** | **0.91 (0.40 to 1.41)** | **0.001** | **0.75 (0.15 to 1.35), p=0.010** |
| **2-Naphthol** |  |  |  |  |
| Nicotine Abstaining^a^ | Ref | Ref | Ref |  |
| Non-tobacco nicotine use | 0.00 (-0.17 to 0.17) | 0.09 (-0.12 to 0.30) | 0.39 | Ref |
| Active smoking | **0.31 (0.05 to 0.58)** | **0.42 (0.14 to 0.69)** | **0.003** | **0.33 (0.00 to 0.65), p=0.048** |
| **CaHEMA** |  |  |  |  |
| Nicotine Abstaining^a^ | Ref | Ref | Ref |  |
| Non-tobacco nicotine use | **0.21 (0.04 to 0.38)** | 0.13 (-0.08 to 0.33) | 0.22 | Ref |
| Active smoking | **0.28 (0.02 to 0.54)** | **0.31 (0.05 to 0.57)** | **0.021** | 0.19 (-0.13 to 0.50), p=0.44 |
| **2HEMA** |  |  |  |  |
| Nicotine Abstaining^a^ | Ref | Ref | Ref |  |
| Non-tobacco nicotine use | -0.14 (-0.30 to 0.02) | -0.09 (-0.31 to 0.12) | 0.39 | Ref |
| Active smoking | 0.18 (-0.63 to 0.43) | 0.24 (-0.04 to 0.52) | 0.087 | **0.34 (0.01 to 0.67), p=0.044** |
| **2CaEMA** |  |  |  |  |
| Nicotine Abstaining^a^ | Ref | Ref | Ref |  |
| Non-tobacco nicotine use | -0.09 (-0.26 to 0.09) | -0.09 (-0.32 to 0.14) | 0.44 | Ref |
| Active smoking | **0.30 (0.03 to 0.57)** | **0.34 (0.04 to 0.63)** | **0.026** | **0.43 (0.08 to 0.78), p=0.012** |
| **34HBMA** |  |  |  |  |
| Nicotine Abstaining^a^ | Ref | Ref | Ref |  |
| Non-tobacco nicotine use | -0.08 (-0.16 to 0.00) | -0.01 (-0.12 to 0.09) | 0.80 | Ref |
| Active smoking | 0.08 (-0.04 to 0.20) | 0.10 (-0.03 to 0.24) | 0.14 | 0.12 (-0.05 to 0.28), p=0.25 |
| **3HPMA** |  |  |  |  |
| Nicotine Abstaining^a^ | Ref | Ref | Ref |  |
| Non-tobacco nicotine use | -0.02 (-0.18 to 0.14) | -0.04 (-0.26 to 0.18) | 0.70 | Ref |
| Active smoking | **0.26 (0.01 to 0.51)** | 0.28 (-0.01 to 0.56) | 0.055 | 0.32 (-0.01 to 0.65), p=0.066 |
| **2CyEMA** |  |  |  |  |
| Nicotine Abstaining^a^ | Ref | Ref | Ref |  |
| Non-tobacco nicotine use | **0.59 (0.32 to 0.79)** | **0.45 (0.41 to 0.75)** | **0.005** | Ref |
| Active smoking | **1.67 (1.30 to 2.03)** | **1.66 (1.26 to 2.05)** | **<0.001** | **1.21 (0.74 to 1.67), p<0.001** |
| **2HPMA** |  |  |  |  |
| Nicotine Abstaining^a^ | Ref | Ref | Ref |  |
| **Non-tobacco nicotine use** | **-0.15 (-0.28 to -0.03)** | -0.06 (-0.23 to 0.11) | 0.48 | Ref |
| Active smoking | 0.10 (-0.10 to 0.30) | 0.17 (-0.05 to 0.39) | 0.13 | 0.2 (-0.03 to 0.5), p=0.1 |
| **1HMPeMA** |  |  |  |  |
| Nicotine Abstaining^a^ | Ref | Ref | Ref |  |
| **Non-tobacco nicotine use** | **0.15 (0.00 to 0.29)** | 0.07 (-0.11 to 0.25) | 0.46 | Ref |
| Active smoking | **0.32 (0.10 to 0.54)** | **0.27 (0.04 to 0.50)** | **0.023** | 0.20 (-0.07 to 0.48), p=0.22 |
| **2HBeMA** |  |  |  |  |
| Nicotine Abstaining^a^ | Ref | Ref | Ref |  |
| Non-tobacco nicotine use | -0.09 (-0.36 to 0.18) | -0.06 (-0.40 to 0.27) | 0.70 | Ref |
| Active smoking | **0.44 (0.02 to 0.86)** | 0.40 (-0.04 to 0.83) | 0.074 | 0.46 (-0.05 to 0.97), p=0.094 |
| **4HBeMA** |  |  |  |  |
| Nicotine Abstaining^a^ | Ref | Ref | Ref |  |
| Non-tobacco nicotine use | -0.15 (-0.31 to 0.02) | -0.07 (-0.29 to 0.15) | 0.55 | Ref |
| Active smoking | 0.25 (-0.01 to 0.51) | **0.31 (0.02 to 0.59)** | **0.036** | **0.37 (0.04 to 0.71), p=0.025** |
| **HPMMA** |  |  |  |  |
| Nicotine Abstaining^a^ | Ref | Ref | Ref |  |
| Non-tobacco nicotine use | -0.01 (-0.12 to 0.10) | 0.05 (-0.09 to 0.20) | 0.45 | Ref |
| Active smoking | 0.15 (-0.03 to 0.31) | **0.18 (0.00 to 0.37)** | **0.049** | 0.13 (-0.09 to 0.35), p=0.44 |
| **PhMA** |  |  |  |  |
| Nicotine Abstaining^a^ | Ref | Ref | Ref |  |
| Non-tobacco nicotine use | -0.07 (-0.20 to 0.06) | 0.02 (-0.16 to 0.19) | 0.87 | Ref |
| Active smoking | -0.02 (-0.22 to 0.18) | 0.06 (-0.17 to 0.29) | 0.61 | 0.04 (-0.23 to 0.31), p=1 |

Abbreviations: FEV1 Forced Expired Volume in first second, FVC Forced Vital Capacity, FEV1/FVC modified Tiffeneau-Pinelli index, FEF 25-75 forced expiratory flow between 25% and 75% of forced vital capacity, LCI Lung Clerance Index LCI, FeNO Fraction of exhaled Nitric Oxide, OHdG 8-Hydroxydesoxyguanosin. NNAL 4-(methylnitrosamino)-1-(3-pyridyl)-1-butanol, 1-OHP 1-Hydroxypyrene (a metabolite of pyrene), 1-Naphthol 1-Hydroxynaphthalene and 2-Naphthol 2-Hydroxynaphthalene (metabolites of naphthalene), **CaHEMA** Glycidamide mercapturic acid (a metabolite of acrylamide), 2HEMA 2-Hydroxyethylmercapturic acid (a metabolite of ethylene oxide, acrylonitrile, or vinyl chloride), 2CaEMA 2-Carbamoylethylmercapturic acid (a metabolite of acrylamide), 34HBMA Dihydroxybutylmercapturic acid (a metabolite of 1,3-butadiene), 3HPMA 3-Hydroxypropylmercapturic acid (a metabolite of acrolein), 2CyEMA 2-Cyanoethylmercapturic acid (a metabolite of acrylonitrile), 2HPMA 2-Hydroxypropylmercapturic acid (a metabolite of propylene oxide), 1HMPeMA, 2HBeMA, 4HBeMA Monohydroxybutenyl-mercapturic acids (metabolites of 1,3-butadiene, 4HBeMA specifically refers to 4-hydroxy-2-buten-1-yl-mercapturic acid), HPMMA 3-Hydroxy-1-methyl-propylmercapturic acid (a metabolite of crotonaldehyde), PhMA Phenylmercapturic acid (a metabolite of benzene). **Bold** if p<0.05 for higher exposures.

Missings: respiratory symptoms are missing in 2 participants (1 nicotine abstaining, 1 occasional using adolescent), spirometry was missing in 1 participant from the daily vaping group. Urinary markers (oxidative stress, PAH, VOC metabolites) are missing in 13 participants (10 nicotine abstaining, 3 occasional using adolescents). No covariables were missing.

a Nicotine abstaining participants had urinary cotinine and NNAL levels below cut-off

b Models were adjusted for sex, age, education, exposure to second-hand smoke, oven / chimney at home, heavy traffic near place of living, any atopic disease.

c p-value come from a t-test, comparing occasional vaping/smoking, daily vaping or daily smoking to nicotine abstaining adolescents (reference group).

d Contrast estimates, corrected 95% confidence interval and p-value comes from a pairwise comparisons of marginal linear predictions, applying Bonferroni correction, comparing daily smoking and daily vaping adolescents.

e FeNO was log transformed as its distribution is right skewed.

f Values (ng/ml) in urine normalized to creatinine concentration (mg/ml) in urine (results in ng/mg creatinine). All PAH and VOC metabolites were log10 transformed to achieve normal distribution.

Appendix Figure 1: Multivariable adjusted models on self-reported and biochemically validated exposure categories and respiratory symptoms, lung function markers (FEV1/FVC, LCI), 8-Hydroxydesoxyguanosin (8-OHdG, oxidative stress marker), 1-Naphthol (PAH metabolite, log10 transformed), and three VOC metabolites (CaHEMA, 2CaEMA, 2CyEMA, all log10 transformed)

Non-using participants reported no vaping or smoking in the past 12 months or never vaping / never smoking but might be exposed to second-hand smoke. Models were adjusted for sex, age, education, exposure to second-hand smoke, oven / chimney at home, heavy traffic near place of living, any atopic disease. p-value come from a t-test, comparing occasional vaping/smoking, daily vaping or daily smoking to non-using adolescents (reference group). Values (ng/ml) in urine normalized to creatinine concentration (mg/ml) in urine (results in ng/mg creatinine). Urinary analyses of oxidative stress, PAH and VOC metabolites were available for 59 participants (19 (66%) non-using, 15 (83%) occasional using, 20 (100%) daily vaping, 5 (100%) daily smoking). Self-reported (nicotine) exposure in blue and self-reported and biochemically validated (nicotine) exposure in red.

# STROBE Statement – Filled Checklist

|  | Item no | Recommendation | Done | Page |
| --- | --- | --- | --- | --- |
| **Title and abstract** | 1 | (*a*) Indicate the study’s design with a commonly used term in the title or the abstract | Yes | 1, 2 |
|  |  | (*b*) Provide in the abstract an informative and balanced summary of what was done and what was found | Yes | 2 |
| Introduction | | |  |  |
| Background/rationale | 2 | Explain the scientific background and rationale for the investigation being reported | Yes | 3 |
| Objectives | 3 | State specific objectives, including any prespecified hypotheses | Yes | 3 |
| Methods | | |  |  |
| Study design | 4 | Present key elements of study design early in the paper | Yes | 3 |
| Setting | 5 | Describe the setting, locations, and relevant dates, including periods of recruitment, exposure, follow-up, and data collection | Yes | 3 |
| Participants | 6 | (*a*) Give the eligibility criteria, and the sources and methods of selection of participants. Describe methods of follow-up | Yes | 3 |
|  |  | (*b*) For matched studies, give matching criteria and number of exposed and unexposed | n/a |  |
| Variables | 7 | Clearly define all outcomes, exposures, predictors, potential confounders, and effect modifiers. Give diagnostic criteria, if applicable | Yes | 4, 5 |
| Data sources/ measurement | 8* | For each variable of interest, give sources of data and details of methods of assessment (measurement). Describe comparability of assessment methods if there is more than one group | Yes | 4, 5 |
| Bias | 9 | Describe any efforts to address potential sources of bias | Yes | 5 |
| Study size | 10 | Explain how the study size was arrived at | Yes | 5 |
| Quantitative variables | 11 | Explain how quantitative variables were handled in the analyses. If applicable, describe which groupings were chosen and why | Yes | 4, 5 |
| Statistical methods | 12 | (*a*) Describe all statistical methods, including those used to control for confounding | Yes | 5 |
|  |  | (*b*) Describe any methods used to examine subgroups and interactions | Yes | 5 |
|  |  | (*c*) Explain how missing data were addressed | Yes | Tables 2 and 3 |
|  |  | (*d*) If applicable, explain how loss to follow-up was addressed | n/a |  |
|  |  | (*e*) Describe any sensitivity analyses | Yes | 5 |
| Results | | |  |  |
| Participants | 13* | (a) Report numbers of individuals at each stage of study—eg numbers potentially eligible, examined for eligibility, confirmed eligible, included in the study, completing follow-up, and analysed | Yes | 6, Figure 1 |
|  |  | (b) Give reasons for non-participation at each stage | n/a |  |
|  |  | (c) Consider use of a flow diagram | Yes | Figure 1 |
| Descriptive data | 14* | (a) Give characteristics of study participants (eg demographic, clinical, social) and information on exposures and potential confounders | Yes | 6, Table 1, Appendix Table 1 |
|  |  | (b) Indicate number of participants with missing data for each variable of interest | Yes | Tables 2 and 3, Appendix Tables 2-6 |
|  |  | (c) Summarise follow-up time (eg, average and total amount) | n/a | Cross-sectional |
| Outcome data | 15* | Report numbers of outcome events or summary measures over time | Yes | 6 |
| Main results | 16 | (*a*) Give unadjusted estimates and, if applicable, confounder-adjusted estimates and their precision (eg, 95% confidence interval). Make clear which confounders were adjusted for and why they were included | Yes | 5, 6 |
|  |  | (*b*) Report category boundaries when continuous variables were categorized | Yes | 6 |
|  |  | (*c*) If relevant, consider translating estimates of relative risk into absolute risk for a meaningful time period | n/a |  |
| Other analyses | 17 | Report other analyses done—eg analyses of subgroups and interactions, and sensitivity analyses | Yes | 6, 7 |
| Discussion | | |  |  |
| Key results | 18 | Summarise key results with reference to study objectives | Yes | 7 |
| Limitations | 19 | Discuss limitations of the study, taking into account sources of potential bias or imprecision. Discuss both direction and magnitude of any potential bias | Yes | 8 |
| Interpretation | 20 | Give a cautious overall interpretation of results considering objectives, limitations, multiplicity of analyses, results from similar studies, and other relevant evidence | Yes | 8 |
| Generalisability | 21 | Discuss the generalisability (external validity) of the study results | Yes | 8 |
| Other information | | |  |  |
| Funding | 22 | Give the source of funding and the role of the funders for the present study and, if applicable, for the original study on which the present article is based | Yes | 1 |

*Give information separately for exposed and unexposed groups.

**Note:** An Explanation and Elaboration article discusses each checklist item and gives methodological background and published examples of transparent reporting. The STROBE checklist is best used in conjunction with this article (freely available on the Web sites of PLoS Medicine at http://www.plosmedicine.org/, Annals of Internal Medicine at http://www.annals.org/, and Epidemiology at http://www.epidem.com/). Information on the STROBE Initiative is available at <http://www.strobe-statement.org>.

# Appendix Reference

1.	Aryal S, Diaz-Guzman E, Mannino DM. Influence of sex on chronic obstructive pulmonary disease risk and treatment outcomes. *Int J Chron Obstruct Pulmon Dis*. 2014;9:1145-54. doi:10.2147/copd.S54476

2.	Hitchman SC, Fong GT, Zanna MP, Thrasher JF, Chung-Hall J, Siahpush M. Socioeconomic status and smokers' number of smoking friends: findings from the International Tobacco Control (ITC) Four Country Survey. *Drug Alcohol Depend*. Oct 1 2014;143:158-66. doi:10.1016/j.drugalcdep.2014.07.019

3.	Raju S, Siddharthan T, McCormack MC. Indoor Air Pollution and Respiratory Health. *Clin Chest Med*. Dec 2020;41(4):825-843. doi:10.1016/j.ccm.2020.08.014

4.	Eisner MD, Jacob P, 3rd, Benowitz NL, Balmes J, Blanc PD. Longer term exposure to secondhand smoke and health outcomes in COPD: impact of urine 4-(methylnitrosamino)-1-(3-pyridyl)-1-butanol. *Nicotine & tobacco research : official journal of the Society for Research on Nicotine and Tobacco*. Aug 2009;11(8):945-53. doi:10.1093/ntr/ntp091

5.	Flor LS, Anderson JA, Ahmad N, et al. Health effects associated with exposure to secondhand smoke: a Burden of Proof study. *Nat Med*. Jan 2024;30(1):149-167. doi:10.1038/s41591-023-02743-4

6.	Lee CH, Chuang HY, Hong CH, et al. Lifetime exposure to cigarette smoking and the development of adult-onset atopic dermatitis. *Br J Dermatol*. Mar 2011;164(3):483-9. doi:10.1111/j.1365-2133.2010.10116.x

7.	Heatherton TF, Kozlowski LT, Frecker RC, Fagerstrom KO. The Fagerstrom Test for Nicotine Dependence: a revision of the Fagerstrom Tolerance Questionnaire. *British journal of addiction*. Sep 1991;86(9):1119-27.

8.	Fillmore MT, Jude R. Defining "binge" drinking as five drinks per occasion or drinking to a .08% BAC: which is more sensitive to risk? *Am J Addict*. Sep-Oct 2011;20(5):468-75. doi:10.1111/j.1521-0391.2011.00156.x

9.	Salem Y, Jakob J, Steinberg R, et al. Cohort Profile Update: The Bern Basel Infant Lung Development Cohort. *International Journal of Epidemiology*. 2023;doi:10.1093/ije/dyad164

10.	van der Velpen V, Liakoni E, Hirt MB, et al. A validated single-step saliva and serum sample extraction LC-MS/MS method for the analysis of nicotine, cotinine and 3’-hydroxycotinine for clinical vaping studies. *Journal of Pharmaceutical and Biomedical Analysis*. 2025/06/15/ 2025;258:116703. doi:<https://doi.org/10.1016/j.jpba.2025.116703>

11.	Sharma P, Sane N, Anand SD, Marimutthu P, Benegal V. Assessment of cotinine in urine and saliva of smokers, passive smokers, and nonsmokers: Method validation using liquid chromatography and mass spectrometry. *Indian J Psychiatry*. May-Jun 2019;61(3):270-276. doi:10.4103/psychiatry.IndianJPsychiatry_61_18

12.	Taylor E, Simonavičius E, McNeill A, et al. Exposure to Tobacco-Specific Nitrosamines Among People Who Vape, Smoke, or do Neither: A Systematic Review and Meta-Analysis. *Nicotine & Tobacco Research*. 2023;26(3):257-269. doi:10.1093/ntr/ntad156

13.	Sambiagio N, Berthet A, Wild P, et al. Associations between urinary biomarkers of oxidative stress and biomarkers of tobacco smoke exposure in smokers. *Sci Total Environ*. Dec 15 2022;852:158361. doi:10.1016/j.scitotenv.2022.158361

14.	Wang Y, Wong LY, Meng L, et al. Urinary concentrations of monohydroxylated polycyclic aromatic hydrocarbons in adults from the U.S. Population Assessment of Tobacco and Health (PATH) Study Wave 1 (2013-2014). *Environ Int*. Feb 2019;123:201-208. doi:10.1016/j.envint.2018.11.068
